# Solvent-Assisted N–O Bond Cleavage and Metal–Metal Bond Formation in the Reduction of Binuclear Nitrosyl Complexes [M_2_Cp_2_(μ-X)(μ‑P*^t^* Bu_2_)(NO)_2_] (MX = MoCl, WI): An Experimental and Theoretical Study

**DOI:** 10.1021/acs.inorgchem.5c03067

**Published:** 2025-08-18

**Authors:** M. Angeles Alvarez, Daniel García-Vivó, Ana M. Guerra, Miguel A. Ruiz

**Affiliations:** Departamento de Química Orgánica e Inorgánica/IUQOEM, 16763 Universidad de Oviedo, Oviedo E-33071, Spain

## Abstract

Reaction of [Mo_2_Cp_2_(μ-Cl)­(μ-P*^t^* Bu_2_)­(NO)_2_] with Na­(Hg) in tetrahydrofuran gave a very air-sensitive species formulated as the oxido–nitrido radical [Mo_2_Cp_2_(N)­(μ-O)­(μ-P*^t^* Bu_2_)­(NO)] according to density functional theory (DFT) calculations, which follows from a fast N–O bond cleavage of a nitrosyl ligand. Reaction of the latter with (NH_4_)­PF_6_ yielded the diamagnetic cation [Mo_2_Cp_2_(μ-N)­(O)­(μ-P*^t^* Bu_2_)­(NO)]^+^ as a mixture of *cis* and *trans* isomers, which display bent-bridging nitrido and terminal oxido ligands, isolated as the corresponding BAr’_4_^–^ salts (Ar’ = 3,5-C_6_H_3_(CF_3_)_2_; Mo–Mo = 2.8365(5) and 2.836(1) Å, respectively). The N–O bond cleavage leading to this radical presumably involves stepwise nitrosyl rearrangements at the dimetal center, first as a solvent-assisted one from terminal to the linear μ-κ_N_:η^2^ coordination mode, then by rearrangements into the μ-κ_N_:κ_O_ and bent μ-κ_O_:η^2^ modes, according to calculations. In contrast, reduction of the related ditungsten complex [W_2_Cp_2_(μ-I)­(μ-P*^t^* Bu_2_)­(NO)_2_] with Na­(Hg) in either tetrahydrofuran or acetonitrile gave the unsaturated anion [W_2_Cp_2_(μ-P*^t^* Bu_2_)­(NO)_2_]^−^ (calcd W–W = 2.601 Å), which upon reaction with (NH_4_)­PF_6_ rendered the hydride-bridged derivative [W_2_Cp_2_(μ-H)­(μ-P*^t^* Bu_2_)­(NO)_2_], an air-sensitive 32-electron complex reacting with CO to give the electron-precise carbonyl derivative [W_2_Cp_2_(H)­(μ-P*^t^* Bu_2_)­(CO)­(NO)_2_], selectively formed with terminal CO and H ligands *cis* and *trans* to the bridging P atom, respectively, and Cp ligands arranged in *syn* conformation.

## Graphical abstract

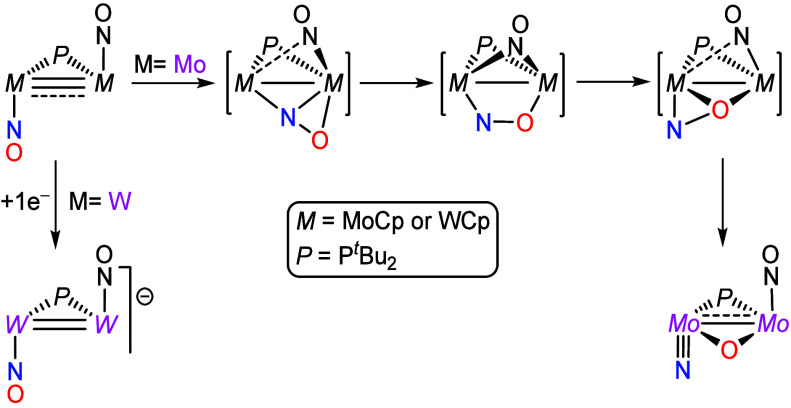

## Introduction

We have shown recently that reduction of the chlorido-bridged dimolybdenum complex [Mo_2_Cp_2_(μ-Cl)­(μ-P*^t^* Bu_2_)­(NO)_2_] (**1a**) with Na­(Hg) in acetonitrile solution triggers, depending on reaction conditions, different activation processes involving coordinated acetonitrile and nitrosyl ligands, with the latter being able to undergo full N–O cleavage and N–C coupling to a molecule of solvent, to eventually yield an acetamidinate complex (Scheme 1).^1^ This is in stark contrast with previous studies on the reduction of the related PPh_2_-bridged ditungsten complex [W_2_Cp_2_(μ-I)­(μ-PPh_2_)­(NO)_2_], which reacts with Na­(Hg) in acetonitrile solution without N–O bond cleavage or solvent coordination to give an unsaturated anion [W_2_Cp_2_(μ-PPh_2_)­(NO)_2_]^−^ having a metal–metal double bond (Scheme 2). The latter gives upon protonation the corresponding hydride-bridged derivative [W_2_Cp_2_(μ-H)­(μ-PPh_2_)­(NO)_2_],^2^ a reactive unsaturated molecule able to induce different E–H bond cleavage and insertion reactions when confronted with *p*-block element molecules.^3,4^

**Scheme 1:**
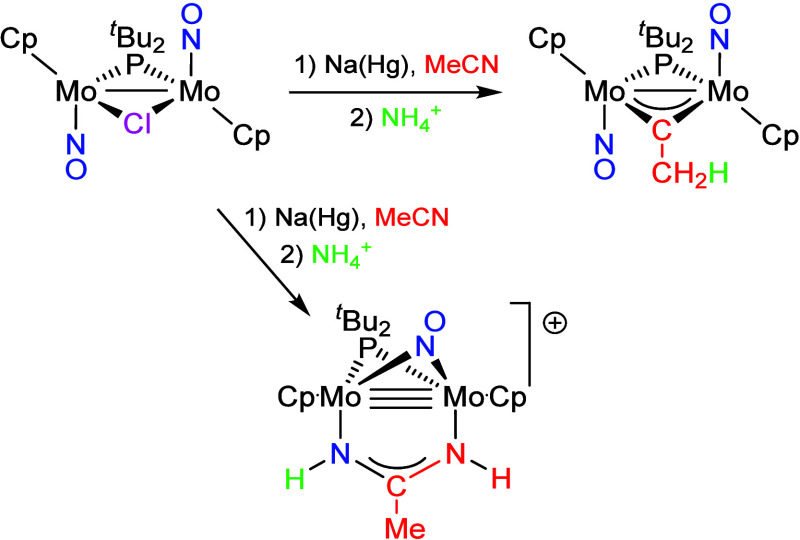
Reduction of Compound **1a** in Acetonitrile Solutions

**Scheme 2:**
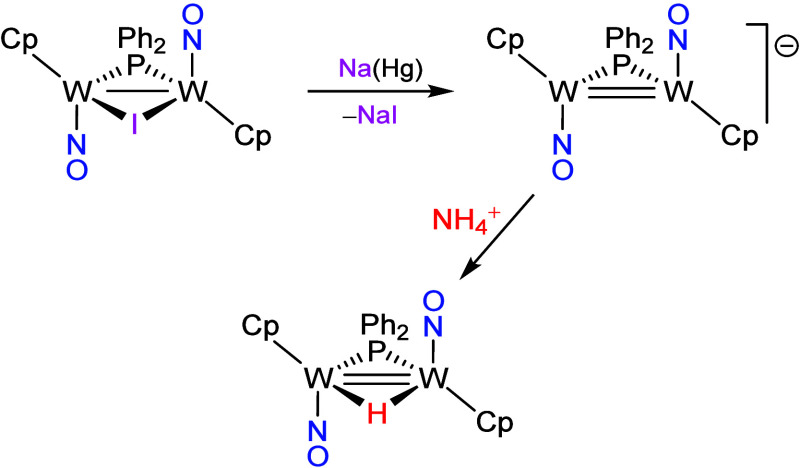
Reduction of [W_2_Cp_2_(μ-I)­(μ-PPh_2_)­(NO)_2_] in Acetonitrile Solutions

The cleavage of the strong N–O bond of nitric oxide when bound to metal centers is a matter of interest not only in the academic context of the coordination chemistry of transition-metal nitrosyl complexes^5^ but also because this elemental process is an essential step in any metal-catalyzed reaction of potential use for the abatement of toxic nitrogen oxide effluents that arise from a variety of large-scale processes (power generation plants, nitric acid production, domestic heating, automotive engines, etc.).^6^ Yet, studies on N–O bond cleavage of the nitrosyl ligand at well-defined binuclear transition-metal complexes are still scarce.^7–10^ This prompted us to further explore our reduction reactions of halide-bridged dinitrosyl complexes to gain knowledge of the influence of the solvent and metal on the final output of these reactions. To this purpose, we have now examined the reduction reactions of the molybdenum complex **1a** using as solvent tetrahydrofuran, a molecule with poorer coordination ability compared to acetonitrile. Moreover, we have also examined similar reduction reactions of the ditungsten analogue [W_2_Cp_2_(μ-I)­(μ-P*^t^* Bu_2_)­(NO)_2_] (**1b**),^11^ which likely would give us information about the influence of metal (Mo vs W) and phosphanyl bridge (P*^t^* Bu_2_ vs PPh_2_) on all these processes. As will be shown below, a delicate balance of both thermodynamic and kinetic factors drives these reduction reactions along two different pathways, with only one of them eventually leading to the oxidative addition of a nitrosyl ligand to the dimetal center. The latter is dominant for the molybdenum complex and involves the active participation of the tetrahydrofuran solvent.

## Results and Discussion

### Reduction of Compound **1a** in Tetrahydrofuran Solutions

Compound **1a** reacts with Na­(Hg) in tetrahydrofuran at room temperature to give brown solutions displaying a strong N–O stretch at 1572 cm^–1^, suggestive of the presence of a neutral (rather than anionic) species and bearing terminal nitrosyl ligands. No ^31^P NMR resonance could be attributed to the major species in these solutions, which is thought to be the 33-electron oxido–nitrido complex [Mo_2_Cp_2_(N)­(μ-O)­(μ-P*^t^* Bu_2_)­(NO)] (**2**) (see below), in any case a quite air-sensitive species that could not be isolated (Scheme 3 and Table 1). Addition of (NH_4_)­PF_6_ to these solutions resulted in oxidation (rather than protonation) of the above paramagnetic complex to yield the diamagnetic cation [Mo_2_Cp_2_(μ-N)­(O)­(μ-P*^t^* Bu_2_)­(NO)]^+^ (**3**) as a ca. 4:1 mixture of *cis* and *trans* isomers, as defined by the relative arrangement of their terminal oxido and nitrosyl ligands. These were more conveniently isolated as the corresponding BAr’_4_^–^ salts (***cis*** **-** and ***trans*** **-3-BAr’**_**4**_) after anion exchange with Na­(BAr’_4_) in dichloromethane solution of the PF_6_^–^ salts initially formed (Ar’ = 3,5-C_6_H_3_(CF_3_)_2_; see the Experimental Section). No other diamagnetic complexes were present in significant amounts in the above reaction mixtures as determined through the corresponding ^31^P NMR spectra. Once the isomers were separated chromatographically from each other, there was no evidence of interconversion between them in solution at room temperature.

**Scheme 3:**
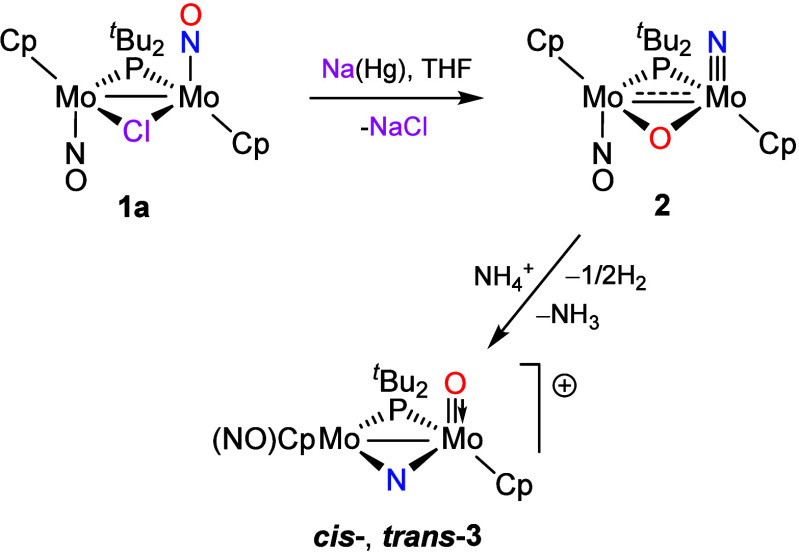
Oxido–Nitrido Derivatives of Compound **1a**

**Table 1:** Selected IR and ^31^P­{^1^H} NMR Data for New Compounds^a^

compound	ν(NO)	δ (P) [*J*_PW_]
[Mo_2_Cp_2_(μ-Cl)(μ-P*^t^* Bu_2_)(NO)_2_] (**1a**)^b^	1602 (w, sh), 1575 (vs)	281.8
[W_2_Cp_2_(μ-I)(μ-P*^t^* Bu_2_)(NO)_2_] (**1b**)^c^	1576 (w, sh), 1555 (vs)	210.1 [334]
[Mo_2_Cp_2_(N)(μ-O)(μ-P*^t^* Bu_2_)(NO)] (**2**)	1572 (vs)^d^	
*cis*-[Mo_2_Cp_2_(μ-N)(O)(μ-P*^t^* Bu_2_)(NO)](BAr’_4_) (***cis*** **-3-BAr’**_**4**_)	1663 (vs)	278.2
*trans*-[Mo_2_Cp_2_(μ-N)(O)(μ-P*^t^* Bu_2_)(NO)](BAr’_4_) (***trans*** **-3-BAr’**_**4**_)	1642 (vs)	267.9
Na[W_2_Cp_2_(μ-P*^t^* Bu_2_)(NO)_2_] (**4**)		284.4 [316]^d^
[W_2_Cp_2_(μ-H)(μ-P*^t^* Bu_2_)(NO)_2_] (**5**)	1574 (w), 1546 (vs)	283.3 [349]^e^
[W_2_Cp_2_(H)(μ-P*^t^* Bu_2_)(CO)(NO)_2_] (**6**)	1953 (vs),^f^ 1644 (s), 1553 (m)	203.8 [323, 164]

a IR spectra recorded in dichloromethane solution, with N–O stretches [ν­(NO)] in cm^–1^; ^31^P­{^1^H} NMR spectra recorded in CD_2_Cl_2_ solution at 121.48 MHz and 293 K, with chemical shifts (δ) in ppm relative to external 85% aqueous H_3_PO_4_ and coupling constants (*J*) in Hz; *J*_PW_ stands for coupling between ^31^P and ^183^W nuclei.

b Data taken from ref 1.

c Data taken from ref 11.

d In tetrahydrofuran solution.

e In C_6_D_6_ solution.

f ν­(CO).

### Structure of Complex **2**

As noted above, the IR spectrum of **2** in tetrahydrofuran solution displays a single N–O stretch at 1572 cm^–1^, only a few cm^–1^ below the strongest band of the parent compound **1a** (Table 1), indicative of its neutral nature and of the presence of terminal nitrosyl ligands. This itself might be consistent with a formulation as [Mo_2_Cp_2_(μ-P*^t^* Bu_2_)­(NO)_2_] (**T**), the neutral radical expectedly resulting from the one-electron reduction of **1a** followed by the release of a chloride anion (eqs 1 and 2). However, this is highly unlikely for an observable species since density functional calculations (DFT, see the Experimental Section) revealed that the Gibbs free energy of such a structure is ca. 25 kcal/mol higher than that of an isomer [Mo_2_Cp_2_(μ-P*^t^* Bu_2_)­(μ-NO)_2_] (**B**) bearing two *bridging* nitrosyls (Figure 1). The latter, however, would display much lower N–O stretches (cf. 1407 cm^–1^ for [Mo_2_Cp_2_(μ-P*^t^* Bu_2_)­(μ-NO)­(NO)_2_])^8a^ and obviously does not match our limited experimental data for **2**.

$$1a+e^{-}\to {\left[1a\right]}^{-}$$ 1

$${\left[1a\right]}^{-}\to T+{\mathrm{Cl}}^{-}$$ 2

**Figure 1:**
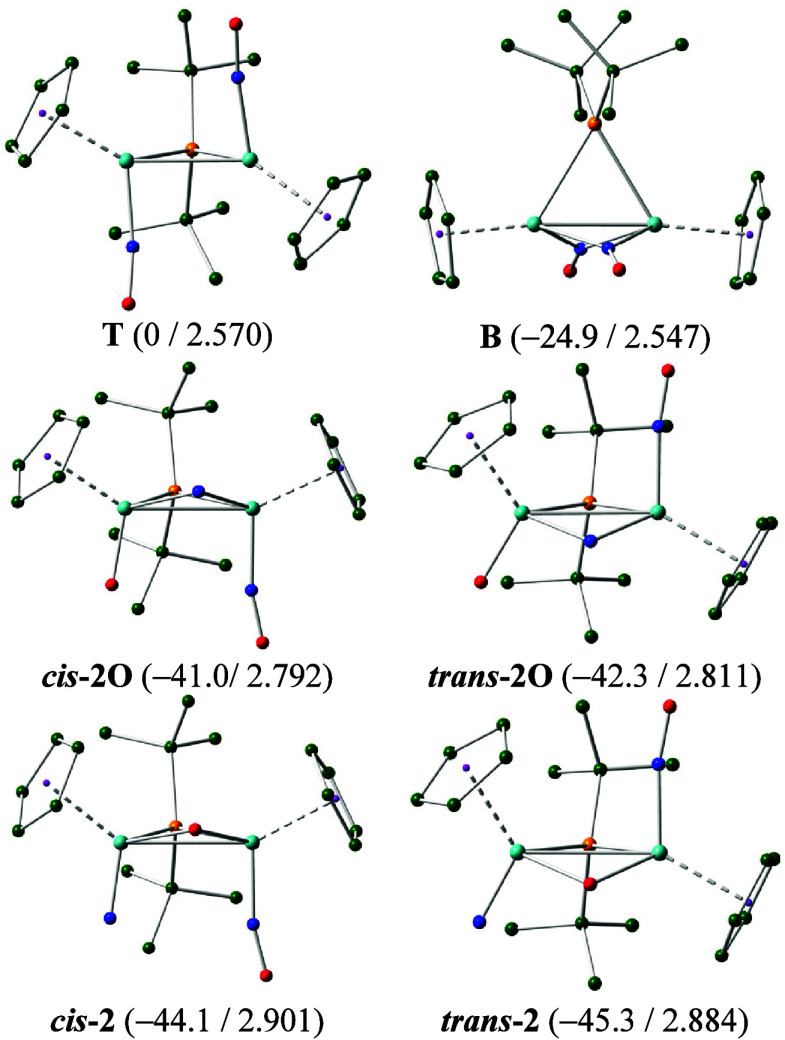
M06L-DFT computed structures of different isomers of dimolybdenum radical **2**, with H atoms omitted for clarity. Color code: Mo (turquoise), P (orange), C (green), N (blue), and O (red). Relative Gibbs free energies in the gas phase at 298 K (in kcal/mol) and intermetallic distances (in Å) indicated between brackets.

By considering the oxido–nitrido nature of the cations **3** derived from **2**, it is sensible to hypothesize that the N–O bond cleavage behind these products might have taken place actually at neutral radical **2**. Indeed, we have computed that different oxido–nitrido formulations for **2** have Gibbs free energies in the range 41–45 kcal/mol below the dinitrosyl structure **T** and well below the nitrosyl-bridged structure **B** too (Figure 1 and the Supporting Information (SI)). Isomers bearing terminal ligands in a relative transoid arrangement were slightly more stable than their cisoid isomers, as expected, and there was a more significant preference of some 3 kcal/mol for the nitrido ligand to be in the terminal coordination mode (Mo–N, ca. 1.67 Å), while the oxido ligand adopts a bridging coordination (Mo–O, ca. 1.96 Å). The energetic difference between the two most stable isomers *trans*- and *cis* **-2** is computed to be only 1.2 kcal/mol in favor of the transoid isomer, so we could not exclude the presence of both isomers in solution. That would be actually consistent with the fact that both *cis* and *trans* isomers of cation **3** are formed upon the reaction of **2** with NH_4_PF_6_, as mentioned above.

The oxidative addition relating the 31-electron dinitrosyl complexes **T** and **B** with their most stable oxido–nitrido isomers **2** provides two additional electrons to the dimetal center, so the latter radicals become 33-electron complexes, for which a metal–metal bond order of 1.5 is to be formulated according to the 18-electron formalism. This is consistent with the strong elongation of the intermetallic bond, from ca. 2.55 Å in either **T** or **B** (formal bond order 2.5) up to some 2.89 Å in the oxido-bridged isomers **2**, a figure approaching single-bond values (cf. 2.905(1) Å in [Mo_2_Cp_2_(μ-P*^t^* Bu_2_)­(μ-NO)­(NO)_2_]).^8a^ Details of the likely elemental steps involved in such an oxidative addition will be discussed later on. We note that previous examples of the full oxidative addition of a nitrosyl ligand at a dimetal center are restricted to the room-temperature rearrangement of the 30-electron nitrosyl-bridged complexes [M_2_Cp*_2_R_2_(μ-NO)_2_] (M_2_ = Mo_2_, MoW; R = CH_2_CMe_3_, CH_2_CMe_2_Ph) to give their oxido–nitrido isomers [M_2_Cp*_2_R_2_(μ-N)­(O)­(NO)], the latter displaying linearly bridging nitrido and terminal oxido ligands and lacking a metal–metal bond.^10i^

### Structure of the Cations *cis*-**3** and *trans*-**3**

The structure of the cation in the BAr’_4_^–^ salt of the minor isomer *trans* **-3** (Figure 2 and Table 2) could be solved and refined satisfactorily even if it displays a whole-body disorder over two close sites in the crystal, with 0.70/0.30 occupancies. The molecule is built from two transoid MoCpL units (L = O, NO) bent-bridged symmetrically by a nitrido ligand (Mo–N ca. 2.13 Å) and asymmetrically bridged by the phosphanido ligand (Mo–P = 2.33(1) and 2.53(1) Å), the latter presumably balancing the different donor contributions of the terminal nitrosyl and oxido ligands. The geometrical parameters for the terminal nitrosyl are as expected; in contrast, we note that the Mo–O length of the terminal oxido ligand, which points away from the dimetal center (Mo–Mo–O = 112.2(3)°), is quite short (1.69(1) Å), thus denoting a significant π-bonding contribution beyond a conventional Mo=O double bond.^7,12^ In the limit of such a contribution, the O atom might be viewed as a four-electron donor, therefore yielding a 34-electron (rather than 32) count at the dimetal center, hence a formal intermetallic bond order of 1 (rather than 2) according to the 18-electron rule (canonical forms ***I*** and ***II*** in Chart 1). The intermetallic distance in cation *trans* **-3** (2.836(1) Å) is substantially longer than the expected figure for a conventional double bond (cf. 2.632(1) Å for [Mo_2_Cp_2_(μ-PCy_2_)­(μ-N=CHPh)­(CO)_2_][Bibr ref13] and actually approaches single-bond figures measured in related molecules (cf. 2.8654(8) Å in [Mo_2_Cp_2_(μ-PCy_2_)­(μ-NH_2_)­(NO)_2_]).^8c^ Thus, it seems that the canonical form ***II*** would better represent the Mo–Mo and Mo–O interactions in this case.

**Figure 2:**
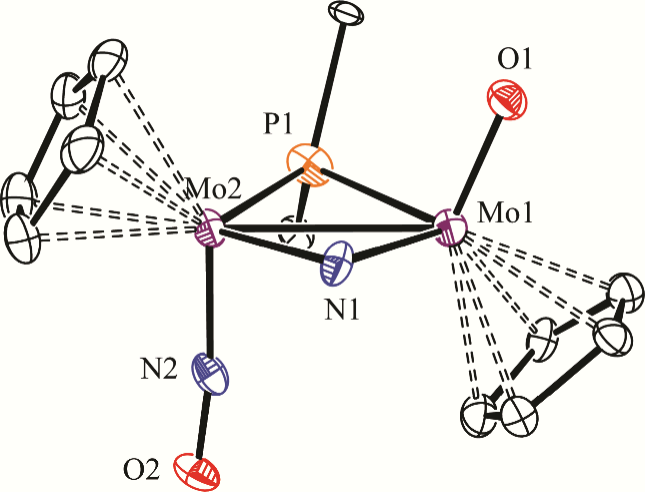
ORTEP diagram (30% probability) of the cation in compound ***trans***-**3-BAr’**_**4**_ with *^t^* Bu (except their C^1^ atoms) and H atoms omitted for clarity (only one of the two disordered cations shown, with 70% occupancy).

**Table 2:** Selected Bond Lengths (Å) and Angles (°) for the Cation in Compound ***trans***-**3-BAr’**_**4**_

Mo1–Mo2	2.836(1)	Mo1–P1–Mo2	71.3(2)
Mo1–P1	2.53(1)	Mo1–N1–Mo2	83.5(4)
Mo1–N1	2.12(1)	Mo1–Mo2–N2	89.6(3)
Mo1–O1	1.69(1)	Mo2–Mo1–O1	112.2(3)
Mo2–P1	2.33(1)	P1–Mo1-Ν1	99.4(4)
Mo2–N1	2.14(1)	P1–Mo1-Ο1	99.2(3)
Mo2–N2	1.79(1)	N1–Mo1-Ο1	102.7(4)
N2–O2	1.22(1)	P1–Mo2-Ν1	105.0(4)
		P1–Mo2-Ν2	97.3(4)
		N1–Mo2-Ν2	89.5(4)

**Chart 1:**
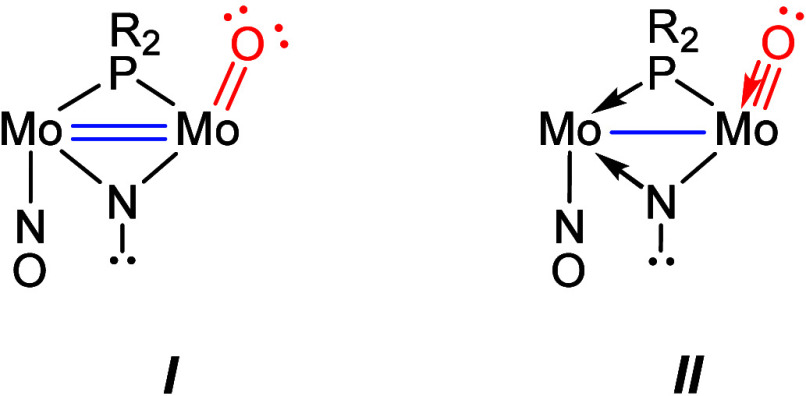
Canonical Forms for the Cation ***trans***-**3**

The angular-bridging coordination of the nitrido ligand in cation ***trans*** **-3** is a bit unexpected since this ligand is more commonly found in terminal or linearly bridging coordination modes^14^ and also because this ligand is most likely terminal in the radical precursor **2**, as discussed above. Examples of crystallographically characterized group 6 metal complexes bearing bent-bridging nitrides are restricted to the family of amidinate complexes [M_2_Cp*_2_(μ-N)_2_(κ^2^-RNCR′NR)_2_] (M = Mo, W; R, R′ = Et, Ph; *^i^* Pr, Me),^15^ with M–N lengths of ca. 1.91 Å, and to the ditungsten alkoxido complex [W_2_(μ-N)_2_(OR)_6_] (R = 2,6-C_6_H_3_ *^i^* Pr_2_), which bears very asymmetric N bridges with W–N lengths of ca. 1.77 and 2.02 Å.^16^ DFT calculations on the cation ***trans*** **-3** and some of their likely isomers (Figure 3 and SI) yield indeed, as the most stable isomer, a transoid structure with a bent-bridging nitrido ligand (Mo–N = 1.858, 1.911 Å; Mo–O = 1.718 Å) akin to its crystallographically determined structure. The corresponding cisoid isomer ***cis*** **-3** is a bit less stable (by 1.6 kcal/mol), possibly because of its higher steric congestion (see below), but isomers derived from the exchange of positions between the nitrido and oxido ligands (that is, with terminal nitrido ligands, ***cis*** **-3N** and ***trans*** **-3N**) were clearly disfavored by some 7 kcal/mol.

**Figure 3:**
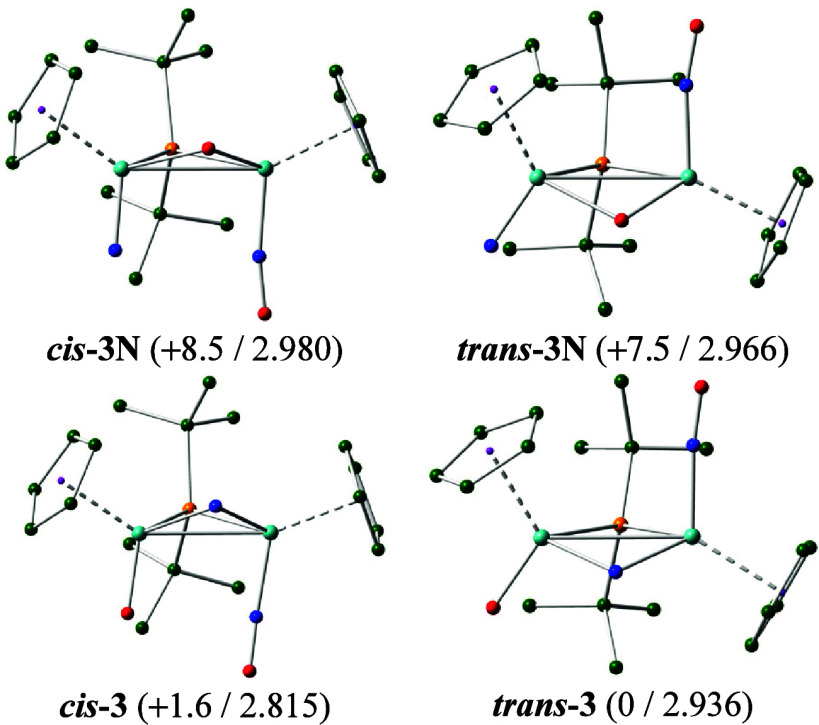
M06L-DFT computed structures of different isomers of the cation **3**, with H atoms omitted for clarity. Color code: Mo (turquoise), P (orange), C (green), N (blue), and O (red). Relative Gibbs free energies in the gas phase at 298 K (kcal/mol) and intermetallic distances (Å) are indicated between brackets.

The structure of the cation in the crystal of the major isomer ***cis*** **-3-BAr’**_**4**_^**–**^ (Figure 4 and Table 3) could also be solved and refined, although the precision of the geometrical parameters so determined was not high, particularly for the terminal oxido and nitrosyl ligands, which were disordered over each other with 0.50 occupancies. Otherwise, the Mo–Mo and Mo–N lengths were analogous to those of cation ***trans*** **-3** discussed above and need no additional comments. We note that the phosphanido ligand in this isomer is now more symmetrical (Mo–P ca. 2.44 and 2.47 Å), a feature also reproduced in its DFT-optimized structure (Mo–P ca. 2.49 and 2.55 Å, Figure 3), therefore not being attributable to disorder in the crystal.

**Figure 4:**
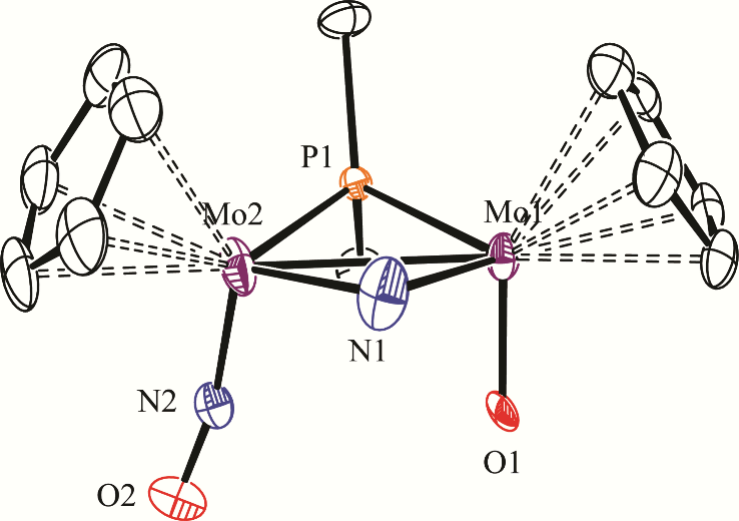
ORTEP diagram (30% probability) of the cation in compound ***cis***-**3-BAr’**_**4**_, with *^t^* Bu (except their C^1^ atoms) and H atoms omitted for clarity (only one of each disordered O and NO sites shown, with 50% occupancy).

**Table 3:** Selected Bond Lengths (Å) and Angles (°) for the Cation in Compound ***cis***-**3-BAr’**_**4**_

Mo1–Mo2	2.8365(5)	Mo1–P1–Mo2	70.56(3)
Mo1–P1	2.444(1)	Mo1–N1–Mo2	86.0(2)
Mo1–N1	2.090(4)	Mo1–Mo2–N2	99.3(7)
Mo1–O1	1.75(1)	Mo2–Mo1–O1	89.9(8)
Mo2–P1	2.467(1)	P1–Mo1−Ν1	100.9(1)
Mo2–N1	2.070(4)	P1–Mo1−Ο1	96.6(11)
Mo2–N2	1.70(1)	N1–Mo1−Ο1	93.8(8)
N2–O2	1.15(1)	P1–Mo2−Ν1	100.7(1)
		P1–Mo2−Ν2	94.3(6)
		N1–Mo2−Ν2	107.1(5)

Spectroscopic data for both isomers of **3-BAr’**_**4**_ are consistent with the structures found in the crystal. In particular, we note the presence in their solid-state IR spectra of a medium-intensity band at 947 (*cis* isomer) or 965 cm^–1^ (*trans*), which we can assign to the Mo–O stretch of the terminal oxido ligand. In solution, the single nitrosyl ligand of these cations displays a N–O stretch that is higher for the *cis* isomer (1663 cm^–1^) than for the *trans* isomer (1642 cm^–1^). This is the same ordering found for the C–O stretch of *cis* and *trans* isomers of different oxido-carbonyl complexes structurally related to cations **3**, such as the PPh_2_-bridged complexes [Mo_2_Cp_2_(μ-PPh_2_)_2_(O)­(CO)][Bibr ref17] or the PCy_2_-bridged complexes [Mo_2_Cp_2_(μ-CPh)­(μ-PCy_2_)­(O)­(CO)].^18^ We also note that one of the *^t^* Bu groups in the *cis* isomer gives rise to two broad ^1^H NMR resonances at 1.98 (6H) and 0.28 (3H) ppm when recorded at room temperature, which appear as three separated resonances at 253 K (δ 2.13, 1.90, and 0.12 ppm; see the Experimental Section and SI). This indicates slow rotation around the corresponding P-CMe_3_ bond on the NMR time scale, a circumstance previously observed for related P*^t^* Bu_2_-bridged cisoid structures (e.g., the *cis* isomer of compound **1a**)^11^ and attributable to the close proximity of one *^t^* Bu group to *both* Cp ligands. Other spectroscopic data for these cations are as expected and deserve no specific comments.

### Reduction Reactions of Compound **1b**

As discussed above, the reactions of the dimolybdenum complex **1a** with Na­(Hg) are quite sensitive to the experimental conditions, particularly to the solvent used (acetonitrile or tetrahydrofuran) (Schemes 1 and 3). This is not the case for ditungsten analogue **1b**, which reacts similarly when using either tetrahydrofuran or acetonitrile to give brown solutions of the Na^+^ salt of the unsaturated anion [W_2_Cp_2_(μ-P*^t^* Bu_2_)­(NO)_2_]^−^ (**4**) (Scheme 4), a process involving the overall transfer of two electrons to the parent complex and release of a iodide anion. This very air-sensitive salt could not be isolated, but it can be protonated readily with (NH_4_)­PF_6_ to give the 32-electron hydride-bridged derivative [W_2_Cp_2_(μ-H)­(μ-P*^t^* Bu_2_)­(NO)_2_] (**5**) as a unique product. The latter still is a quite air-sensitive product, yet it can be isolated as a purple solid. Its unsaturated nature was tested through its reaction with carbon monoxide (1 atm), which takes place in a few minutes at room temperature to give the corresponding electron-precise carbonyl derivative [W_2_Cp_2_(H)­(μ-P*^t^* Bu_2_)­(CO)­(NO)_2_] (**6**). This behavior is parallel to that of the related PPh_2_-bridged derivative of [W_2_Cp_2_(μ-I)­(μ-PPh_2_)­(NO)_2_] (Scheme 2),^2^ except for the stereochemistry of compound **6** (vide infra). However, the general stability of the P*^t^* Bu_2_-bridged ditungsten products was significantly lower, likely due to the increased electron density that the *^t^* Bu groups induce at the dimetal center, and these were not further investigated.

**Scheme 4:**
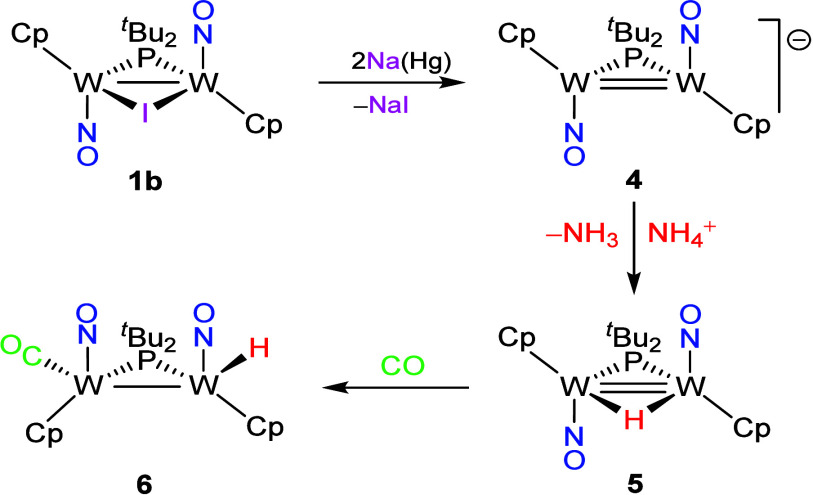
Formation and Derivatives of Anion **4** ^a^

a Anion **4** is formed as the corresponding Na^+^ salt.

### Structure of Anion **4**

Due to the high air-sensitivity of anion **4**, we were not able to identify its N–O stretches in solution. However, we could record its ^31^P NMR spectrum, which displays a resonance at 284.4 ppm equally coupled to both W atoms, indicating the chemical equivalence of both metal fragments. The one-bond ^31^P–^183^W coupling of 316 Hz for **4** is somewhat lower than the one measured for its PPh_2_-bridged analogue (360 Hz),^2^ as expected from the presence of less electronegative *^t^* Bu substituents (compared to Ph groups) at the P atom.^19^ All of these suggest that anion **4** would display a comparable symmetrical structure with terminal rather than bridging nitrosyl ligands. To further support that assumption, we performed DFT calculations on both possible isomers of **4** (Figure 5 and SI) to find that the gas-phase structure with terminal nitrosyls indeed has a Gibbs free energy some 6.5 kcal/mol lower than that of its NO-bridged isomer [W_2_Cp_2_(μ-P*^t^* Bu_2_)­(μ-NO)_2_]^−^ (**4-B**). We note that this difference was even higher for the related PPh_2_-bridged complex (15.8 kcal/mol).^2^ Since, in any case, these are the reverse orderings than the one found for the dimolybdenum radicals **T** and **B** discussed above, we performed analogous calculations on the hypothetical dimolybdenum anion [Mo_2_Cp_2_(μ-P*^t^* Bu_2_)­(NO)_2_]^−^ (isomers **Mo-T** and **Mo-B**) to find that, in this case, the energetic difference almost vanishes out. Thus, we conclude that, in this family of unsaturated anions, the tungsten atoms favor the structure with terminal rather than bridging nitrosyl ligands, whereas the presence of the *^t^* Bu substituents at the P atom seems to disfavor the structure with terminal nitrosyls, likely for steric reasons.

**Figure 5:**
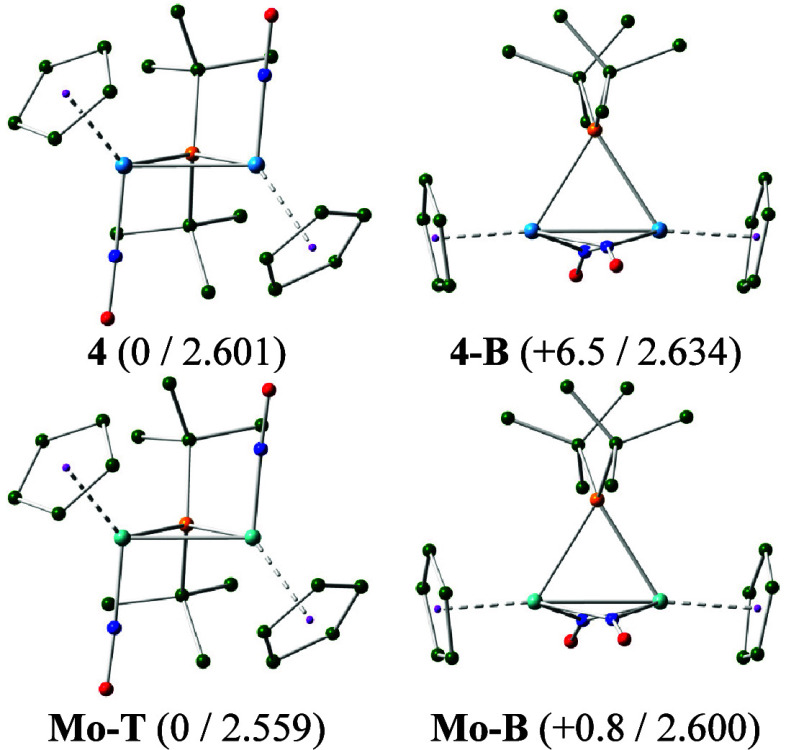
M06L-DFT computed structures of anion **4**, its NO-bridged isomer **4-B**, and their dimolybdenum analogues, with H atoms omitted for clarity. Color code: Mo/W (turquoise), P (orange), C (green), N (blue), and O (red). Relative Gibbs free energies in the gas phase at 298 K (in kcal/mol) and intermetallic distances (in Å) indicated between brackets.

### Structure of the Hydride Derivatives **5** and **6**

The IR spectrum of the unsaturated hydride **5** in solution displays two N–O stretches at 1574 and 1546 cm^–1^, just some 5 cm^–1^ lower than those of the parent iodide-bridged complex **1b**, which is consistent with the retention of terminal nitrosyls, while their relative intensity (weak and strong, in order of decreasing frequencies) is indicative of their antiparallel arrangement.^20,21^ The bridging hydride ligand gives rise to a shielded ^1^H NMR resonance at −10.66 ppm, strongly coupled to both ^183^W nuclei (*J*_HW_ = 147 Hz). These spectroscopic features are very similar to those of its PPh_2_-bridged analogue (δ = −10.79 ppm; *J*_HW_ = 145 Hz), the structure of which was determined through an X-ray study (W–W = 2.7699(7) Å). The absence of a measurable two-bond P–H coupling in these complexes can be attributed to the transoid positioning of PR_2_ and H ligands in these molecules (P–W–H ca. 100° in the PPh_2_ complex).^22^ According to DFT calculations on the latter complex, the double metal–metal bond to be formally proposed for these molecules according to the 18-electron rule should be more precisely described as composed of a bicentric W_2_ σ interaction and a closed tricentric W_2_H interaction.^2^

Compound **5** gives rise to a strongly deshielded ^31^P resonance at 283.3 ppm, a chemical shift comparable to that of anion **4** but much higher than that of its electron-precise iodide-bridged precursor **1b**. Besides this, its coupling to the equivalent ^183^W nuclei (*J*_PW_ = 348 Hz) is anomalously high. By assuming a dependence of ^1^ *J*_PW_ with Pauling′s electronegativity (χ_P_) of the second donor atom at the bridging site similar to that found for related PPh_2_-bridged complexes (42 Hz per unit of χ_P_)^3^ and by considering the experimental value of 334 Hz for ^1^ *J*_PW_ in the iodide complex **1b** (χ_P_ = 2.66 for I), we could estimate a dependence of ca. ^1^ *J*_PW_ = 222 + 42χ_P_ for the general family of P*^t^* Bu_2_-bridged complexes *trans*-[W_2_Cp_2_(μ-X)­(μ-P*^t^* Bu_2_)­(NO)_2_]. That would yield an expected P–W coupling of 314 Hz for the hydride-bridged complex **5** (χ_P_ = 2.20 for H), which is 34 Hz below the experimental value of 348 Hz. A similar difference was found for the PPh_2_-bridged hydride complex akin to **5**, and this is taken as another indication of the different electronic structure derived from the one-electron donor nature of the bridging hydrogen atom as compared to bridging ligands acting as three-electron donors (halogens, SR, PR_2_, etc.).^4^

The IR spectrum of complex **6** in solution displays a very strong C–O stretch at 1953 cm^–1^ corresponding to a terminal carbonyl and two N–O stretches corresponding to terminal nitrosyls with relative intensities (strong and medium, in order of decreasing frequencies) indicative of a mutual cisoid arrangement. This stretching pattern is very similar to that of the iodide complex [W_2_Cp_2_I­(μ-P*^t^* Bu_2_)­(CO)­(NO)_2_], a molecule displaying Cp ligands placed at the same side of the W_2_P plane (*syn* conformation), with the carbonyl ligand being positioned *cis* relative to the P atom and the terminal iodide *trans* to it, as substantiated through an X-ray study.^11^ A similar structure is proposed for **6**, with just replacing the iodine atom in the mentioned structure with an H atom. In agreement with this proposal, the hydride ligand in **6** gives rise to a ^1^H NMR resonance (δ −1.85 ppm) much less shielded than that of **5**, indicating its terminal coordination now, while its very low coupling of just 4 Hz to the ^31^P nucleus reveals a transoid positioning relative to the phosphanido ligand.^22^ Moreover, the unique carbonyl ligand of the molecule gives rise to a ^13^C NMR resonance at 223.5 ppm, with a relatively large two-bond P–C coupling of 15 Hz, which indicates *cis* positioning relative to the P atom. Evidence of the *syn* arrangement of the Cp ligands in **6** stems first from the observation of a very broad ^1^H NMR resonance for one of the *^t^* Bu groups of the molecule. As noted above, this is a spectroscopic feature commonly found for related P*^t^* Bu_2_-bridged complexes bearing Cp ligands in a *syn* (or cisoid) conformation,^11^ as this forces their close proximity to one of the *^t^* Bu substituents of the phosphanido ligand, which causes a slowing down of the rotation that usually averages the corresponding Me resonances. In agreement with this, on lowering of the temperature, the very broad *^t^* Bu resonance at ca. 1.2 ppm for **6** eventually splits into three resonances, appearing at 1.80, 1.55, and 0.15 ppm at 193 K (there is also splitting of the resonance of the other *^t^* Bu group). Besides this, a NOESY spectrum of **6** recorded at this temperature revealed significant NOE enhancements between the most shielded Me resonance of this *^t^* Bu group and *both* Cp ligands (see SI), which is only possible in a *syn* arrangement of the latter ligands relative to the W_2_P plane.

We should note that the stereochemistry of **6** is different from that of its PPh_2_-bridged analogue,^2^ as judged from their substantially distinct IR patterns and P–W couplings (cf. *J*_PW_ = 323, 164 Hz for **6** but *J*_PW_ = 304, 299 Hz for its PPh_2_-bridged analogue). We have not investigated the origin of this difference, but it seems unlikely that a sterically crowded *syn* isomer as **6** would be the most stable out of the eight possible isomers for complexes of type [M_2_Cp_2_X­(μ-PR_2_)­(CO)­(NO)_2_].^11^ We rather trust that **6** is a kinetic product, with its steric crowding perhaps hindering its rearrangement into a thermodynamically more stable isomer.

### Pathways for the N–O Bond Cleavage Leading to Radical **2**

In their study on the mentioned rearrangement of the nitrosyl-bridged complexes [M_2_Cp*_2_R_2_(μ-NO)_2_] (M_2_ = Mo_2_, MoW) to give their oxido–nitrido isomers [M_2_Cp*_2_R_2_(μ-N)­(O)­(NO)], Legzdins and co-workers proposed that the pertinent N–O bond cleavage might take place via an intermediate species in which one of the nitrosyl ligands adopts the rare five-electron-donor linear μ-κ_N_:η^2^ coordination mode.^10i^ In line with this idea, we have recently shown that the linearly κ_N_:η^2^-bridged nitrosyl complex [W_2_Cp_2_(μ-P*^t^* Bu_2_)­(CO)­(μ-κ_N_:η^2^-NO)­(NO)]^+^ indeed can render an oxido–nitrido derivative, although this requires visible-UV excitation.^7^ To explore a possible involvement of this and related unusual coordination modes of the nitrosyl ligand in the formation of the oxido–nitrido radical **2**, we have performed DFT calculations on likely intermediates and transition states connecting the structure of the dinitrosyl radical [Mo_2_Cp_2_(μ-P*^t^* Bu_2_)­(NO)_2_] (**T**) (presumably formed first in the reduction of **1a**; eqs 1 and 2) with its most stable oxido–nitrido isomer ***trans***-**2**, unveiling a relatively complex and multistep reaction pathway (Figure 6 and SI).

**Figure 6:**
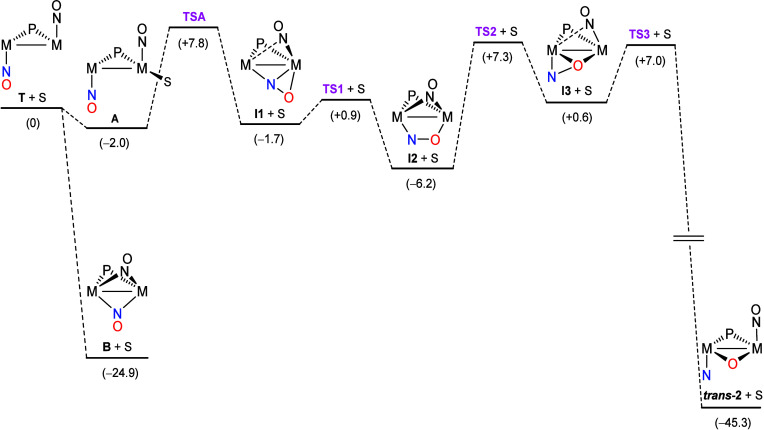
M06L-DFT computed reaction pathway for the formation of the oxido–nitrido radical **2** (M = Mo; S = tetrahydrofuran; bond orders not indicated in drawings). Relative Gibbs free energies in the gas phase at 298 K (kcal/mol) are indicated between brackets.

Our calculations indicate that an intermediate with a linear μ-κ_N_:η^2^-NO ligand (**I1**, N–O = 1.264 Å) would also display a semibridging coordination of the second nitrosyl ligand and would be just slightly more stable (by 1.7 kcal/mol) than the initial radical **T**. The linear μ-κ_N_:η^2^-NO ligand might then rearrange almost barrierless through **TS1** (just 2.6 kcal/mol above **I1**) into the μ-κ_N_:κ_O_-NO coordination mode, with an elongated N–O bond (formally double) of 1.304 Å, while the second nitrosyl ligand adopts a conventional κ_N_:κ_N_-NO bridging coordination mode (N–O = 1.234 Å), thus yielding the more stable intermediate **I2** (6.2 kcal/mol below **T**). To our knowledge, there is no previous example of a polynuclear complex exhibiting a bridging nitrosyl with such a coordination mode, but the related μ_4_-κ_N_:κ_N_:κ_O_:κ_O_ mode has been previously substantiated in the hexanuclear anion [{Re_3_(μ-H)_3_(CO)_10_}_2_(μ_4_-NO)]^−^.^23^ The rearrangement of the κ_N_:κ_O_-bridging nitrosyl would continue by the approach of its oxygen atom to the second metal center (through **TS2**, 13.5 kcal/mol above **I2**), thus rendering a third intermediate species **I3** of about the same energy as the parent **T** but now having a bent μ-κ_O_:η^2^-NO nitrosyl with the O atom at the bridging position and a quite elongated N–O bond (1.417 Å), in an alkenyl-like coordination mode neither identified previously in a complex. We note that the rearrangement involved in the sequence **I1** → **I2** → **I3**, whereby the N and O atoms roughly exchange their coordination sites, is somehow reminiscent of the nitrosyl/isonitrosyl rearrangement M–NO → M­(η^2^-NO) → M–ON that can take place at mononuclear complexes under photochemical activation.^24,25^ Intermediate **I3** would finally evolve readily through **TS3** (just 6.4 kcal/mol above **I3**) with full cleavage of the N–O bond to render bridging oxido and terminal nitrido ligands as present in ***trans*** **-2**. The estimated activation barrier for the overall **T** → ***trans*** **-2** isomerization would be given by the **I2**/**TS2** gap, which is 13.5 kcal/mol, a modest figure consistent with a rearrangement taking place very rapidly at room or even low temperatures, in agreement with experiment. But the latter would only apply if radical **T** could evolve directly to intermediate **I1**. However, if radical **T** first isomerized into the much more stable NO-bridged form **B**, then the evolution from **B** to ***trans*** **-2** would have a kinetic barrier of at least 32.2 kcal/mol (corresponding to the **B**/**TS2** gap) and certainly would not take place rapidly at room temperature. Since we failed to find a transition state connecting the structures **T** and **I1**, we are forced to hypothesize that the formation of the relatively stable nitrosyl-bridged isomer **B** has to be somehow blocked to allow for the fast and irreversible N–O bond cleavage process to take place first directly from **T**. By recalling that reductions of **1a** with Na­(Hg) in acetonitrile invariably involve coordination of a molecule of solvent (Scheme 1),^1^ then we propose that tetrahydrofuran (S) might coordinate reversibly to radical **T**, thus avoiding an almost certainly irreversible isomerization into the NO-bridged form **B**. DFT calculations indicate that an adduct [Mo_2_Cp_2_(μ-P*^t^* Bu_2_)­(NO)_2_(S)] (**A**) with the added solvent molecule S *trans* to the P atom is just 2 kcal/mol below the Gibbs free energy of the reagents (**T** + S), thus indicating a very weak coordination of the solvent. Yet, the formation of adduct **A** enables bypassing the rearrangement into the nitrosyl-bridged **B** without significant modification of the kinetics of the overall process since the dissociative transition state **TSA** connecting this adduct to intermediate **I1** and free solvent is only 9.8 kcal/mol above **A** (Figure 6), so the kinetic barrier for the solvent-assisted overall process from **T** to ***trans*** **-2** would still be given by the **I2**/**TS2** gap and then expected to be very fast at room temperature, as noted above.

## Concluding Remarks

Reduction of [Mo_2_Cp_2_(μ-Cl)­(μ-P*^t^* Bu_2_)­(NO)_2_] with Na­(Hg) in a tetrahydrofuran solution proceeds through one-electron transfer and chloride release to presumably yield the neutral 31-electron radical [Mo_2_Cp_2_(μ-P*^t^* Bu_2_)­(NO)_2_]. In this poorly coordinating solvent, the latter dinitrosyl complex presumably undergoes fast oxidative addition of a nitrosyl ligand to yield the oxido–nitrido radical [Mo_2_Cp_2_(N)­(μ-O)­(μ-P*^t^* Bu_2_)­(NO)] in a solvent-assisted and largely exergonic process, resulting in the formation of terminal nitrido and bridging oxido ligands, according to DFT calculations. Protonation of this species results in an overall one-electron oxidation to yield the diamagnetic cation [Mo_2_Cp_2_(μ-N)­(O)­(μ-P*^t^* Bu_2_)­(NO)]^+^ as a mixture of *cis* and *trans* isomers, which however display bridging nitrido and terminal oxido ligands, more stable than their corresponding (N)­(μ-O) isomers. The fast N–O bond cleavage leading to the oxido–nitrido radical presumably involves stepwise nitrosyl rearrangement at the dimetal center from the terminal to different bridging coordination modes, starting with a solvent-assisted rearrangement into the linear μ-κ_N_:η^2^ mode that bypasses the formation of the nitrosyl-bridged isomer [Mo_2_Cp_2_(μ-P*^t^* Bu_2_)­(μ-NO)_2_], a much more stable molecule that would otherwise act as a kinetic well. The μ-κ_N_:η^2^ intermediate then would undergo sequential N–O bond-weakening rearrangements into novel bridging modes μ-κ_N_:κ_O_ and bent μ-κ_O_:η^2^ before the N–O bond cleavage eventually takes place, according to DFT calculations, which also predict a low kinetic barrier of just 13.5 kcal/mol for the overall process. In contrast to this behavior, the reduction of the related ditungsten complex [W_2_Cp_2_(μ-I)­(μ-P*^t^* Bu_2_)­(NO)_2_] with Na­(Hg) proceeds through a two-electron transfer in either tetrahydrofuran or acetonitrile solutions to give the unsaturated anion [W_2_Cp_2_(μ-P*^t^* Bu_2_)­(NO)_2_]^−^, which would display terminal rather than bridging nitrosyl ligands, an effect attributable to the presence of W (instead of Mo) atoms in the anion, according to calculations, thus overrunning the effect of the steric pressure introduced by the bulky *^t^* Bu substituents, which disfavors the structure with terminal nitrosyls. Protonation of the latter anion expectedly gives the hydride-bridged derivative [W_2_Cp_2_(μ-H)­(μ-P*^t^* Bu_2_)­(NO)_2_], a quite air-sensitive and electron-deficient complex rapidly reacting with CO at room temperature to give the corresponding electron-precise carbonyl derivative [W_2_Cp_2_(H)­(μ-P*^t^* Bu_2_)­(CO)­(NO)_2_], selectively formed as one of the eight possible isomers.

## Experimental Section

### General Procedures and Starting Materials

General experimental procedures, as well as the preparation of compounds [Mo_2_Cp_2_(μ-Cl)­(μ-P*^t^* Bu_2_)­(NO)_2_] (**1a**)^1^ and [W_2_Cp_2_(μ-I)­(μ-P*^t^* Bu_2_)­(NO)_2_] (**1b**),^11^ have been described previously (Cp = η^5^-C_5_H_5_). Na­(BAr’_4_) and all other reagents were obtained from commercial suppliers (Ar’ = 3,5-C_6_H_3_(CF_3_)_2_). IR stretching frequencies of NO and CO ligands are given in wavenumber units (cm^–1^). NMR chemical shifts (δ) are given in ppm, relative to internal tetramethylsilane (^1^H, ^13^C) or external 85% aqueous H_3_PO_4_ solutions (^31^P), and coupling constants (*J*) are given in Hz.

### Safety Statement

No uncommon hazards should be noted in the preparations described below.

### Preparation of Tetrahydrofuran Solutions of [Mo_2_Cp_2_(N)­(μ-O)­(μ-P*^t^* Bu_2_)­(NO)] (**2**)

A solution of compound **1a** (0.040 g, 0.071 mmol) in tetrahydrofuran (THF) (8 mL) was stirred with excess Na­(Hg) (ca. 1 mL of a 0.5% amalgam) for 20 min at room temperature to give a brown solution that was filtered using a cannula. The resulting very air-sensitive solution contained **2** as the major nitrosyl-containing product, as judged from IR spectroscopy, likely as a mixture of *cis* and *trans* isomers (see discussion), and it was used without further purification.

### Preparation of ***cis***- and ***trans***-[Mo_2_Cp_2_(μ-N)­(O)­(μ-P*^t^* Bu_2_)­(NO)]­(BAr’_4_) (***cis***- and ***trans***-**3-BAr’_4_**)

Solid (NH_4_)­PF_6_ (0.035 g, 0.215 mmol) was added to a filtered THF solution of compound **2**, prepared as described above (ca. 0.071 mmol), and the mixture was stirred at room temperature for 5 min to give a brown solution containing a ca. 4:1 mixture of the *cis* (δ_P_ 272.6) and *trans* (δ_P_ 263.9) isomers of compound **3-PF**_**6**_, as determined by ^31^P NMR spectroscopy. The solvent was then removed under a vacuum, the residue was extracted with dichloromethane, and the extracts were filtered to give, after removal of solvent, a crude mixture of isomers *cis*- and *trans* of salt **3-PF**_**6**_ as a brown solid (0.020 g, ca. 0.033 mmol, 46%). Solid Na­(BAr’_4_) (0.030 g, 0.034 mmol) and dichloromethane (8 mL) were then added to this residue, and the mixture was stirred for 5 min and filtered again to yield the corresponding mixture of isomers *cis* and *trans* of salt **3-BAr’**_**4**_, which could be separated chromatographically. To this purpose, the solvent was first removed from the above solution, the residue was extracted with dichloromethane/petroleum ether (1/1), and the extracts were chromatographed on alumina IV at 253 K. Elution with dichloromethane/petroleum ether (4/1) gave a minor brown fraction, yielding, upon removal of solvents, compound ***trans*** **-3-BAr’**_**4**_ as a brown solid (0.005 g, 5%). Elution with neat dichloromethane gave a major orange-brown fraction, yielding, upon removal of solvents, compound ***cis*** **-3-BAr’**_**4**_ as a brown solid (0.018 g, 18%). The crystals used in the X-ray study of these isomers were grown in both cases through the slow diffusion of layers of toluene and petroleum ether into concentrated dichloromethane solutions of the compounds at 253 K. Data for isomer ***cis*-3-BAr’** **_4_**: Anal. Calcd for C_50_H_40_BF_24_Mo_2_N_2_O_2_P: C, 43.19; H, 2.90; N, 2.01. Found: C, 42.80; H, 3.64; N, 2.19. IR (Nujol): 947 (Mo–O). ^1^H NMR (400.13 MHz, CD_2_Cl_2_, 295 K): δ 7.72 (s, br, 8H, Ar’), 7.56 (s, 4H, Ar’), 6.31, 6.02 (2s, 2 × 5H, Cp), 1.98 (br, 6H, Me), 1.54 (d, *J*_HP_ = 17, 9H, *^t^* Bu), 0.28 (vbr, 3H, Me). ^1^H NMR (400.13 MHz, CD_2_Cl_2_, 253 K): δ 7.72 (s, br, 8H, Ar’), 7.56 (s, 4H, Ar’), 6.33, 6.04 (2s, 2 × 5H, Cp), 2.13 (d, *J*_HP_ = 12, 3H, Me),1.90 (d, *J*_HP_ = 14, 3H, Me), 1.53 (d, *J*_HP_ = 17, 9H, *^t^* Bu), 0.12 (d, *J*_HP_ = 19, 3H, Me). ^13^C­{^1^H} NMR (100.63 MHz, CD_2_Cl_2_, 233 K): δ 161.6 [q, *J*_C11B_ = 49, C^1^(C_6_H_3_)], 135.6 [s, C^2^(C_6_H_3_)], 128.5 [qq, *J*_CF_ = 30, *J*_C11B_ = 5, C^3^(C_6_H_3_)], 124.4 (q, *J*_CF_ = 274, CF_3_), 117.5 [sept, *J*_CF_ = 4, C^4^(C_6_H_3_)], 103.9, 98.9 (2s, Cp), 49.4 [d, *J*_CP_ = 7, C^1^(*^t^* Bu)], 43.0 [d, *J*_CP_ = 8, C^1^(*^t^* Bu)], 34.1 [d, *J*_CP_ = 11, Me], 33.5 [d, *J*_CP_ = 4, Me], 32.7 [d, *J*_CP_ = 4, C^2^(*^t^* Bu)], 31.8 [s, Me]. Data for isomer ***trans*-3-BAr’** **_4_**. IR (Nujol): 956 (Mo–O). ^1^H NMR (400.13 MHz, CD_2_Cl_2_, 295 K): δ 7.72 (s, br, 8H, Ar’), 7.56 (s, 4H, Ar’), 6.33, 6.04 (2s, 2 × 5H, Cp), 1.61 (d, *J*_HP_ = 16, 9H, *^t^* Bu), 1.39 (d, br, *J*_HP_ = 16, 9H, *^t^* Bu).

### Preparation of Solutions of Na­[W_2_Cp_2_(μ-P*^t^* Bu_2_)­(NO)_2_] (**4-Na**)

A solution of compound **1b** (0.040 g, 0.048 mmol) in either tetrahydrofuran or acetonitrile (8 mL) was stirred with excess Na­(Hg) (ca. 0.5 mL of a 0.5% amalgam) for 15 min at room temperature to give a brown solution that was filtered using a cannula. The resulting very air-sensitive solution contained **4-Na** as the major product, as judged from ^31^P NMR spectroscopy, and was used without further purification.

### Preparation of [W_2_Cp_2_(μ-H)­(μ-P*^t^* Bu_2_)­(NO)_2_] (**5**)

A tetrahydrofuran or acetonitrile solution of compound **4-Na**, prepared as described above (ca. 0.048 mmol), was filtered over solid (NH_4_)­PF_6_ (0.024 g, 0.147 mmol), and the mixture was stirred at room temperature for 5 min to give a purple solution. After removal of the solvent, the residue was extracted with toluene (2 × 5 mL), and the extracts were filtered over diatomaceous earth. Removal of the solvent from the filtrate gave essentially pure compound **5** as a purple solid (0.015 g, 44%). Further purification (at the cost of partial decomposition) was achieved upon chromatography on alumina IV at 253 K (elution with dichloromethane/petroleum ether 3/1) to yield pure **5** as a quite air-sensitive purple solid. ^1^H NMR (300.13 MHz, C_6_D_6_): δ 5.46 (s, 10H, Cp), 1.42 (d, *J*_HP_ = 14, 18H, *^t^* Bu), −10.66 (s, *J*_HW_ = 147, 1H, μ-H).

### Preparation of [W_2_Cp_2_H­(μ-P*^t^* Bu_2_)­(CO)­(NO)_2_] (**6**)

Carbon monoxide was gently bubbled for 1 min through a crude tetrahydrofuran solution of compound **5**, prepared in situ as described above from **1b** (0.040 g, 0.048 mmol), and stirring was continued for a further 10 min to yield a yellow-brown solution. After removal of the solvent from this solution, the residue was extracted with dichloromethane/petroleum ether (1/2), and the extracts were chromatographed on alumina IV at 253 K. Elution with dichloromethane/petroleum ether (4/1) gave a yellow fraction, yielding, after removal of solvents, compound **6** as a yellow solid (0.012 g, 34%). Anal. Calcd for C_20_H_31_Cl_2_N_2_O_3_PW_2_ (**6**·CH_2_Cl_2_): C, 29.40; H, 3.82; N, 3.43. Found: C, 29.19; H, 3.60; N, 3.12. ^1^H NMR (400.13 MHz, CD_2_Cl_2_, 295 K): δ 5.66, 5.59 (2s, 2 × 5H, Cp), 1.50 (d, *J*_HP_ = 15, 9H, *^t^* Bu), 1.2 (vbr, 9H, *^t^* Bu), −1.85 (d, *J*_HP_ = 4, *J*_HW_ = 119, 1H, W–H). ^1^H NMR (400.13 MHz, CD_2_Cl_2_, 193 K): δ 5.75, 5.66 (2s, 2 × 5H, Cp), 1.91 (s, br, 3H, Me), 1.80 (d, *J*_HP_ = 12, 3H, Me), 1.55 (d, *J*_HP_ = 11, 3H, Me), 1.24 (d, *J*_HP_ = 11, 3H, Me), 0.88 (d, *J*_HP_ = 10, 3H, Me), 0.14 (d, *J*_HP_ = 18, 3H, Me). ^13^C­{^1^H} NMR (100.63 MHz, CD_2_Cl_2_, 295 K): δ 223.5 (d, *J*_CP_ = 15, WCO), 93.5, 92.3 (2s, Cp), 48.4 [d, *J*_CP_ = 17, C^1^(*^t^* Bu)], 41.8 [d, *J*_CP_ = 13, C^1^(*^t^* Bu)], 33.4 [s, br, C^2^(*^t^* Bu)], 33.2 [d, *J*_CP_ = 4, C^2^(*^t^* Bu)].

### X-ray Structure Determination of Compounds ***cis***- and ***trans***-**3-BAr’_4_**

Data collection for these compounds was performed at ca. 100 K on a Rigaku XtaLAB Synergy-S Flow diffractometer using Cu Kα radiation. The data collection strategy was defined with the software CrysAlisPro system (CCD 43.122a 64-bit),^26^ and the CrysAlisPro 1.171.43.122a software was used for data reduction.^26^ Numerical absorption correction based on Gaussian integration over a multifaceted crystal model and empirical absorption correction using spherical harmonics were applied using the SCALE2 ABSPACK scaling algorithm as implemented in the CrysAlisPro system. Using the program suite WinGX,^27^ the structures were solved using SIR92 ^28^ or SHELXL2018/3 ^29^ and refined with full-matrix least-squares on *F* ^2^ using the latter program. In general, all nonhydrogen atoms were refined anisotropically, except for atoms involved in disorder, with the latter causing the appearance of B-level alerts in the corresponding checkcif file, and all hydrogen atoms were positioned geometrically and refined using a riding model. In compound ***cis*** **-3-BAr’**_**4**_, the terminal NO and O ligands in the cation were found to be disordered over each other and were satisfactorily modeled with 0.5 occupancies. The identification of the single atom bridging the molybdenum atoms, as nitrogen rather than oxygen, was ascertained since this assignment led to more sensible thermal parameters for such a bridging ligand. There is a weak contact of ca. 2.7 Å between the bridging N atom in the cation and one of the two sites of the O­(nitrosyl) atom of its closest cation in the crystal lattice, possibly imposed by crystal packing, causing the appearance of a B-level alert in the corresponding checkcif file. On the other hand, two CF_3_ groups of the anion were rotationally disordered and satisfactorily refined over two sites each with 0.5 occupancies; nevertheless, the EADP instruction had to be applied on one of these groups to achieve a satisfactory model. After convergence, the strongest residual peaks (2.45–1.8 eÅ^–3^) were placed around one of the disordered CF_3_ groups. Compound ***trans*** **-3-BAr’**_**4**_ crystallized with one dichloromethane molecule, and the whole cation was disordered over two sites and satisfactorily refined with 0.7/0.3 occupancies. Several CF_3_ groups of the anion were also rotationally disordered, four of which were satisfactorily refined over two sites, each with 0.7/0.3 occupancies. After convergence, the strongest residual peaks (1.89–1.38 eÅ^–3^) were close to the solvent molecule and CF_3_ groups.

### Computational Details

DFT calculations were carried out using the GAUSSIAN16 package, the M06L functional, and 6-31G* basis for light elements (P, N, O, C, and H) as described previously.^1^ SDD effective core potentials and its associated basis set were used for Mo and W atoms.^30^ Transition states were searched for by using either Synchronous Transit-Guided Quasi-Newton (STQN) methodologies (keywords QST2 or QST3) or the “distinguished reaction coordination procedure” by choosing an internal coordinate (typically a distance or angle) as the reaction coordinate and running an energy scan calculation along it, with the maxima of this plot being then used as the starting point for a conventional transition-state optimization. Frequency analysis was performed for all the stationary points to ensure that either a minimum structure with no imaginary frequencies or a saddle point with only one negative frequency along the reaction coordinate was achieved. This calculation also provides thermochemical information about the reaction pathways at 298.15 K and 1 atm using a harmonic approximation. The connectivity of the optimized transition states was corroborated in the forward and backward direction via IRC calculations or by manual displacement of the geometrical parameters along the negative frequency and further optimization of the resulting geometries.

## Supplementary Material





## Notes

Crystal data for compounds ***cis-*** and ***trans**-* **3-BAr’**_**4**_ (CCDC 2466925 and 2466926), IR and NMR spectra for all new compounds, and results of DFT calculations (PDF)
Cartesian coordinates of all DFT-computed species (XYZ)
