# Supplementary material for: Solvent-Assisted N–O Bond Cleavage and Metal–Metal Bond Formation in the Reduction of Binuclear Nitrosyl Complexes [M2Cp2(μ-X)(μ‑P t Bu2)(NO)2] (MX = MoCl, WI): An Experimental and Theoretical Study
Source: Inorg Chem. 2025 Aug 18;64(34):17513–22. doi: 10.1021/acs.inorgchem.5c03067 (PMC12406184; doi:10.1021/acs.inorgchem.5c03067)
Supplement: Supplementary file 1 [file ic5c03067_si_001.pdf]

Supporting Information

Solvent Assisted N–O Bond Cleavage and Metal-Metal Bond Formation in the Reduction of Binuclear Nitrosyl Complexes [$M_2Cp_2(\mu$-X)(μ-$P^tBu_2)(NO)_2$] (MX = MoCl, WI). An Experimental and Theoretical Study.

M. Angeles Alvarez, Daniel García-Vivó,* Ana M. Guerra, and Miguel A. Ruiz*

Departamento de Química Orgánica e Inorgánica/IUQOEM, Universidad de Oviedo, E-33071 Oviedo, Spain.

Corresponding Author E-mail: garciavdaniel@uniovi.es (D.G.V.), mara@uniovi.es (M.A.R).

Table S1. Crystal Data for New Compounds

	***cis*-3-BAr'$_4$**	***trans*-3-BAr'$_4$**$\cdot CH_2Cl_2$
mol formula	$C_{50}H_{39}BF_{24}Mo_2N_2O_2P$	$C_{51}H_{42}BCl_2F_{24}Mo_2N_2O_2P$
mol wt	1389.49	1475.42
cryst syst	monoclinic	monoclinic
space group	$P2_1$/c	$P2_1$/c
radiation (λ, Å)	1.54184	1.54184
a, Å	12.96620(4)	13.14120(10)
b, Å	17.64805(6)	24.8458(3)
c, Å	23.62590(8)	17.7888(2)
α, deg	90	90
β, deg	97.3780(3)	104.2950(10)
γ, deg	90	90
V, Å^3	5361.51(3)	5628.27(11)
Z	4	4
calcd density, g cm^{-3}	1.721	1.741
absorp coeff, mm^{-1}	5.263	5.907
temperature, K	100.0(1)	100.0(1)
θ range (deg)	3.14/70.08	3.12/72.13
index ranges (h, k, l)	−15, 15; −21, 21 −28, 27	−12, 15; −30, 30 −21, 21
no. of reflns collected	270449	136572
no. of indep reflns (R_{int})	10161 (0.0434)	11002 (0.0639)
reflns with $I > 2\sigma(I)$	9501	9145
R indexes [data with $I > 2\sigma(I)$]^a	R_1 = 0.0502 wR_2 = 0.1218^b	R_1 = 0.0870 wR_2 = 0.2362^c
R indexes (all data)^a	R_1 = 0.0527 wR_2 = 0.1236^b	R_1 = 0.1014 wR_2 = 0.2463^c
GOF	1.027	1.136
no. of restraints/params	4 / 763	0 / 953
$\Delta\rho$(max., min.), eÅ^{-3}	2.414 / −1.876	1.894 / −1.357
CCDC deposition no	2466925	2466926

^{a} $R = \Sigma||F_o| - |F_c|| / \Sigma|F_o|$. $wR = [\Sigma w(|F_o|^2 - |F_c|^2)^2 / \Sigma w|F_o|^2]^{1/2}$. $w = 1/[\sigma^2(F_o^2) + (aP)^2 + bP]$ where $P = (F_o^2 + 2F_c^2)/3$. ^{b} a = 0.0534, b = 20.2577. ^{c} a = 0.0893, b = 49.3753.

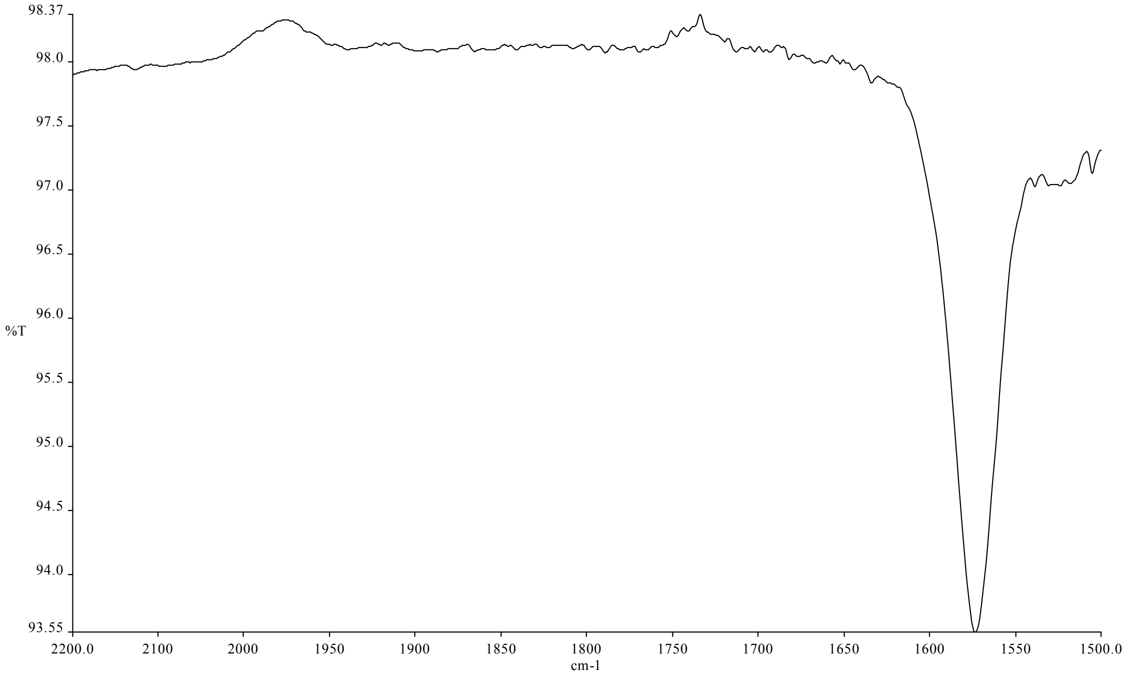

Figure S1. IR spectrum of compound **2** in tetrahydrofuran solution.

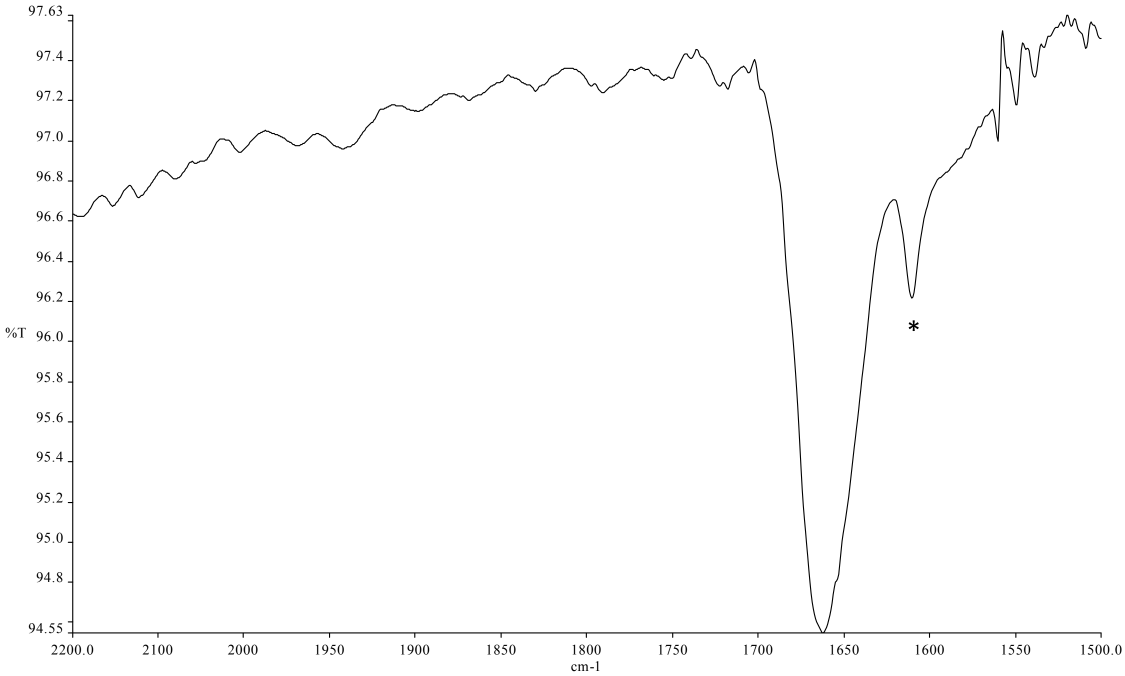

Figure S2. IR spectrum of compound ***cis*-3-BAr'$_4$** in dichloromethane solution (* = ν(C–C), Ar')

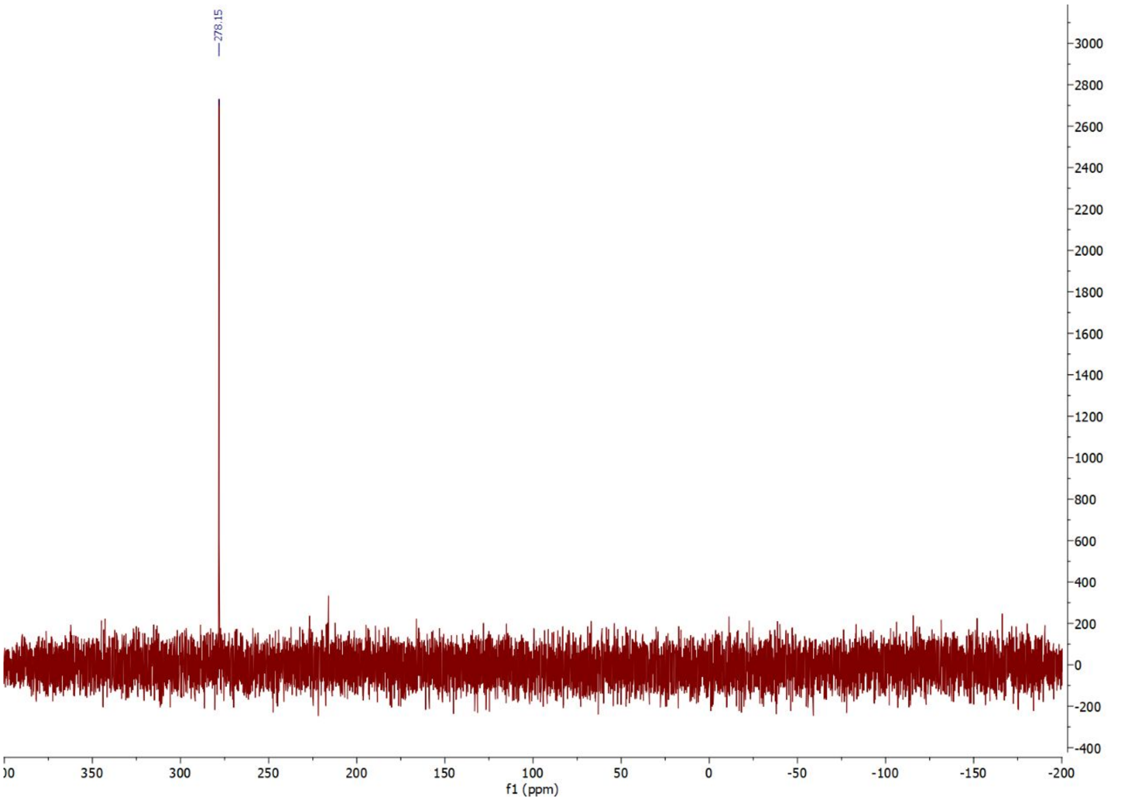

Figure S3. $^{31}P\{^{1}H\}$ NMR spectrum of compound ***cis*-3-BAr'$_4$** (CD_2Cl_2).

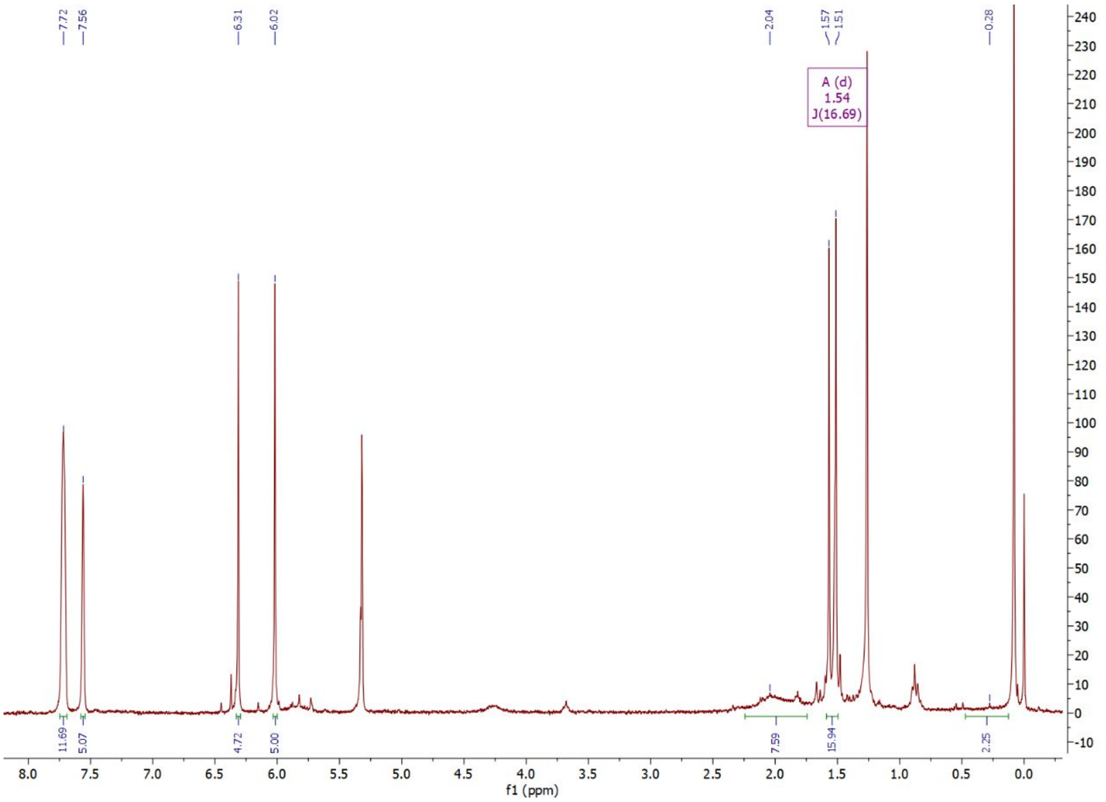

Figure S4. ^{1}H NMR spectrum of compound ***cis*-3-BAr'$_4$** (CD_2Cl_2, 295 K).

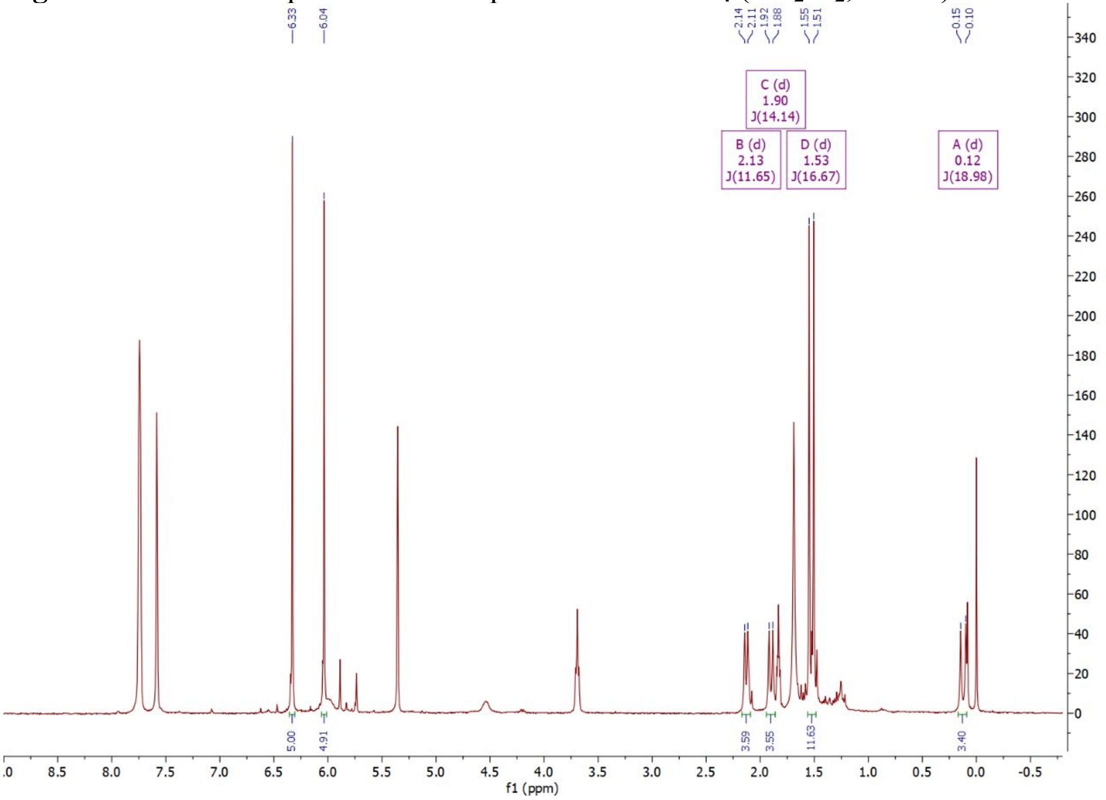

Figure S5. ^{1}H NMR spectrum of compound ***cis*-3-BAr'$_4$** (CD_2Cl_2, 253 K).

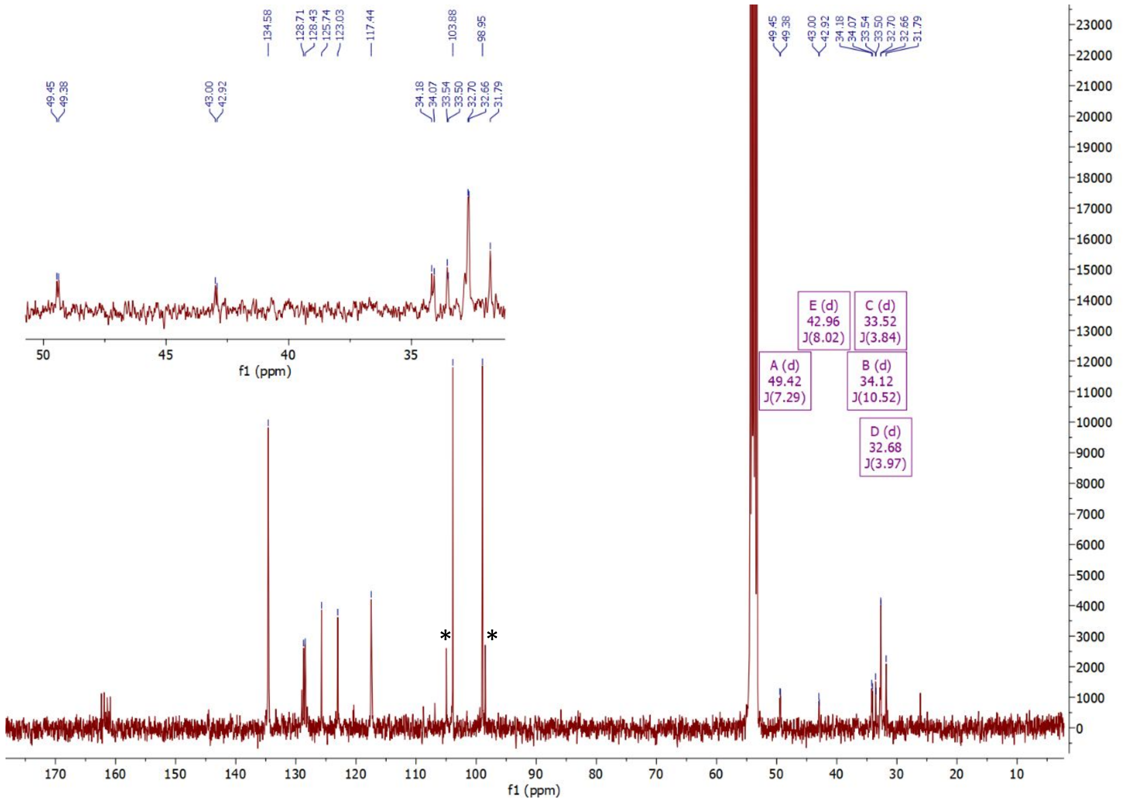

Figure S6. $^{13}C\{^{1}H\}$ NMR spectrum of compound ***cis*-3-BAr'$_4$** (CD_2Cl_2, 233 K). Resonances marked with * correspond to residual ***trans*-3-BAr'$_4$** in the sample.

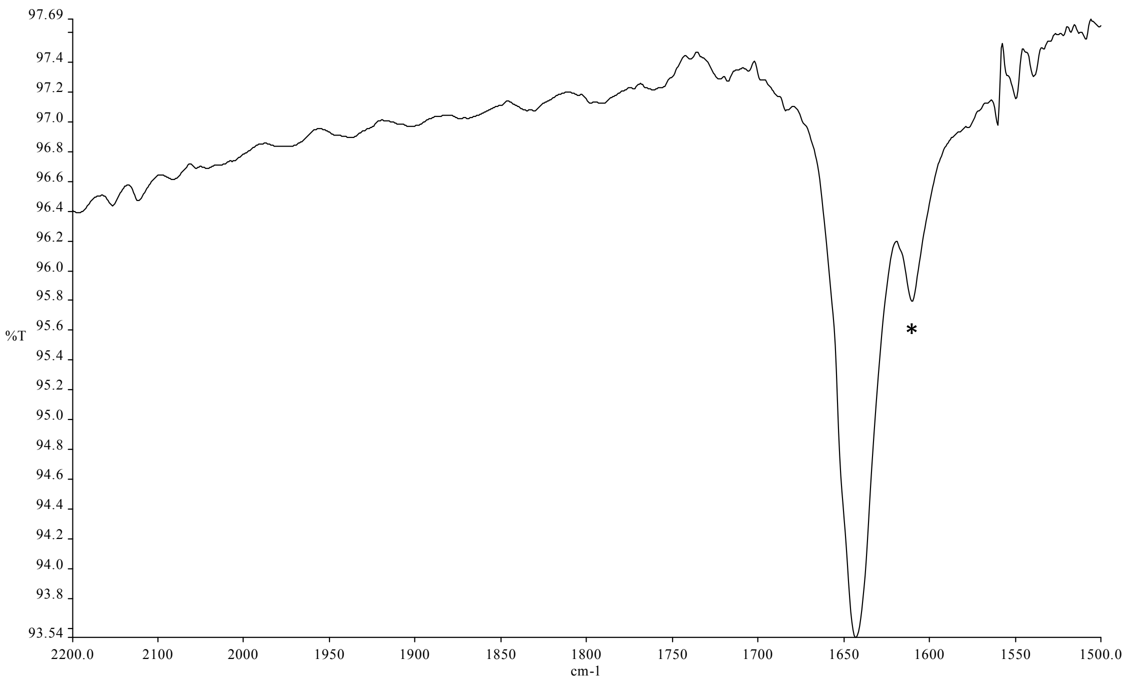

Figure S7. IR spectrum of compound ***trans*-3-BAr'$_4$** in dichloromethane solution (* = ν(C–C), Ar').

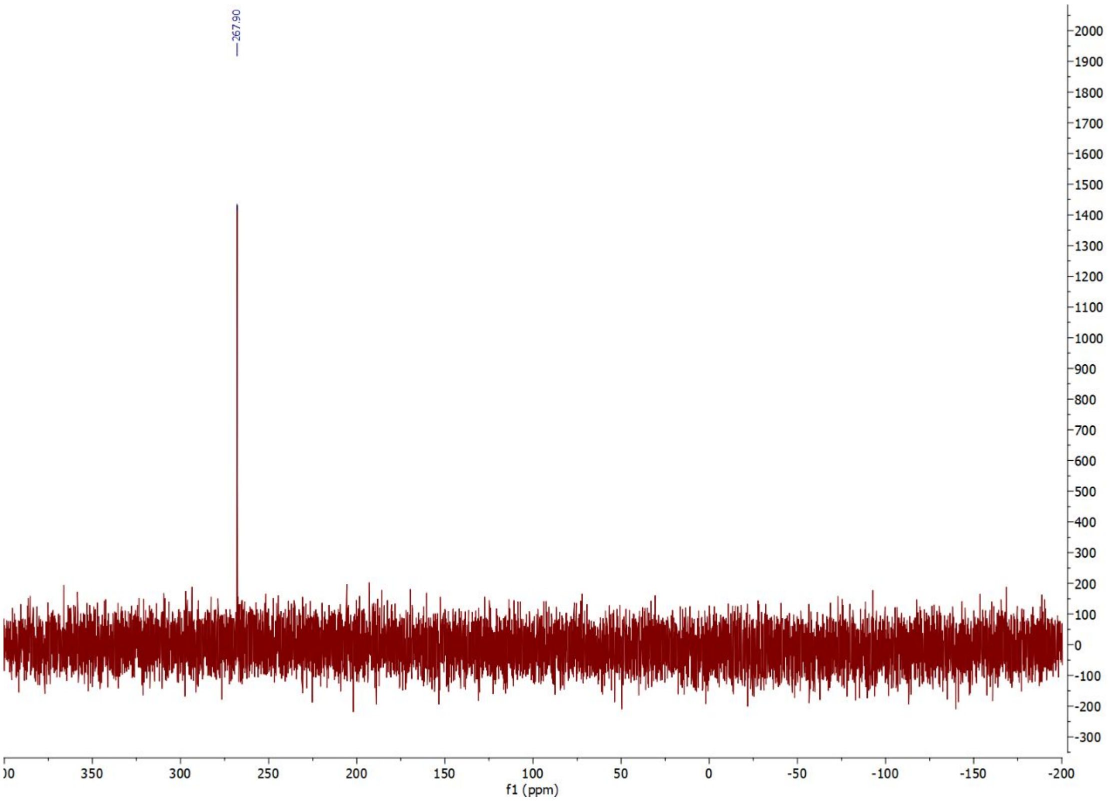

Figure S8. $^{31}P\{^{1}H\}$ NMR spectrum of compound ***trans*-3-BAr'$_4$** (CD_2Cl_2).

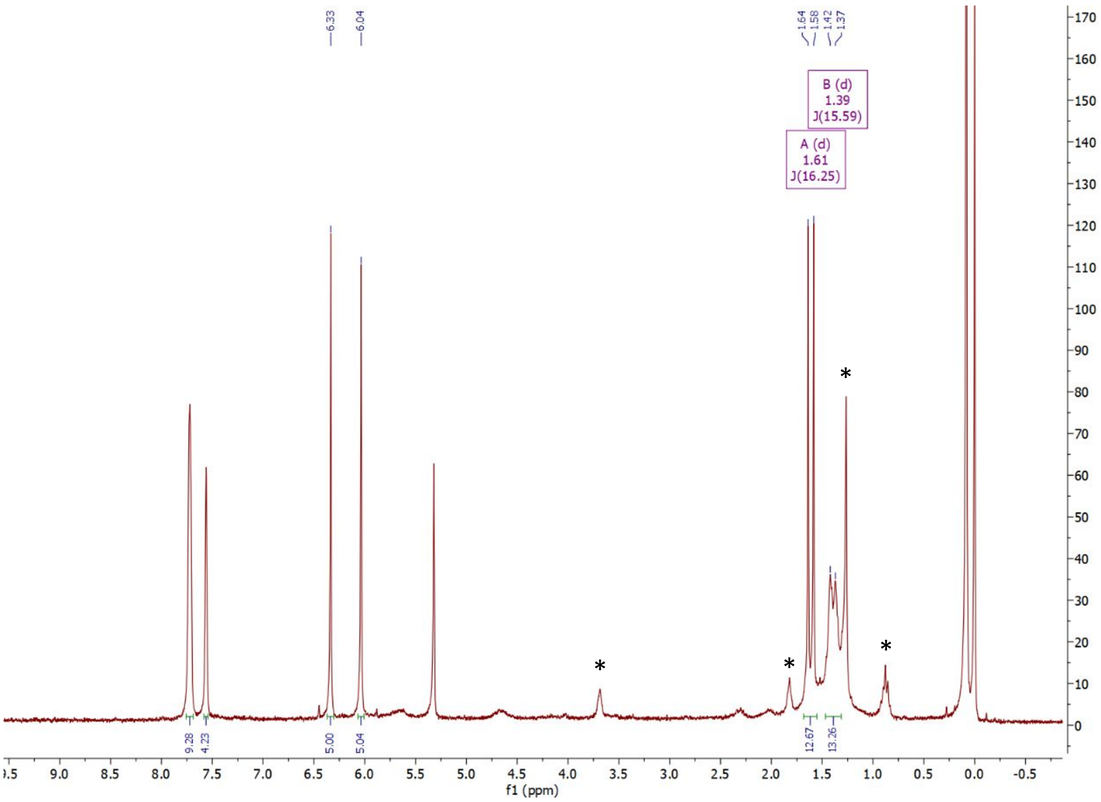

Figure S9. ^{1}H NMR spectrum of compound ***trans*-3-BAr'$_4$** (CD_2Cl_2). Resonances marked with * correspond to residual solvents (petroleum ether, tetrahydrofuran) in the sample.

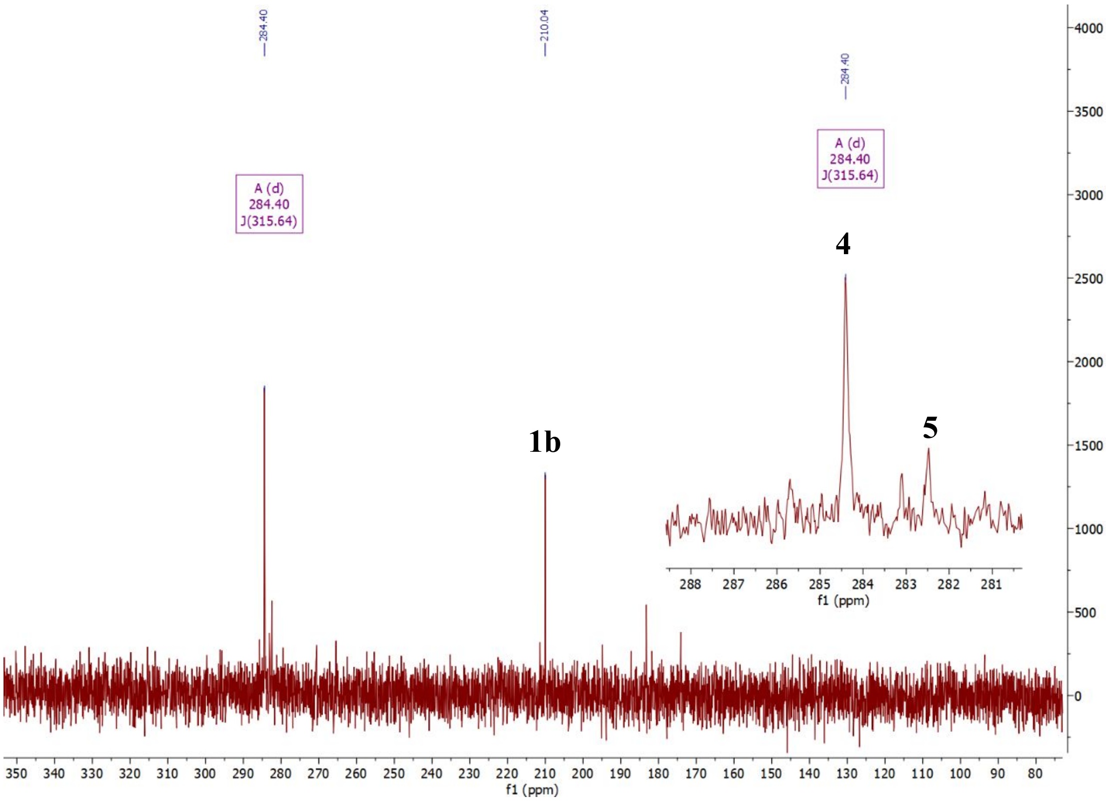

Figure S10. $^{31}P\{^{1}H\}$ NMR spectrum of compound **4-Na** in tetrahydrofuran solution. The minor resonance at 282.5 ppm corresponds to a small amount of hydride **5** formed during manipulation, and that at 210.0 ppm corresponds to residual parent complex **1b** in the sample.

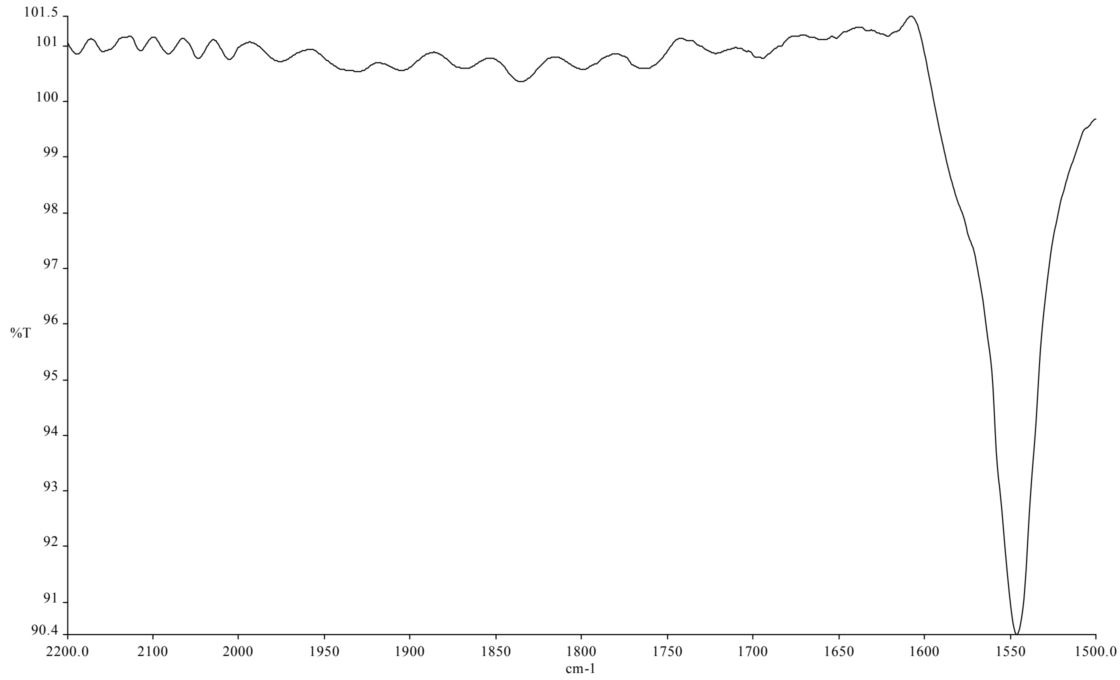

Figure S11. IR spectrum of compound **5** in dichloromethane solution.

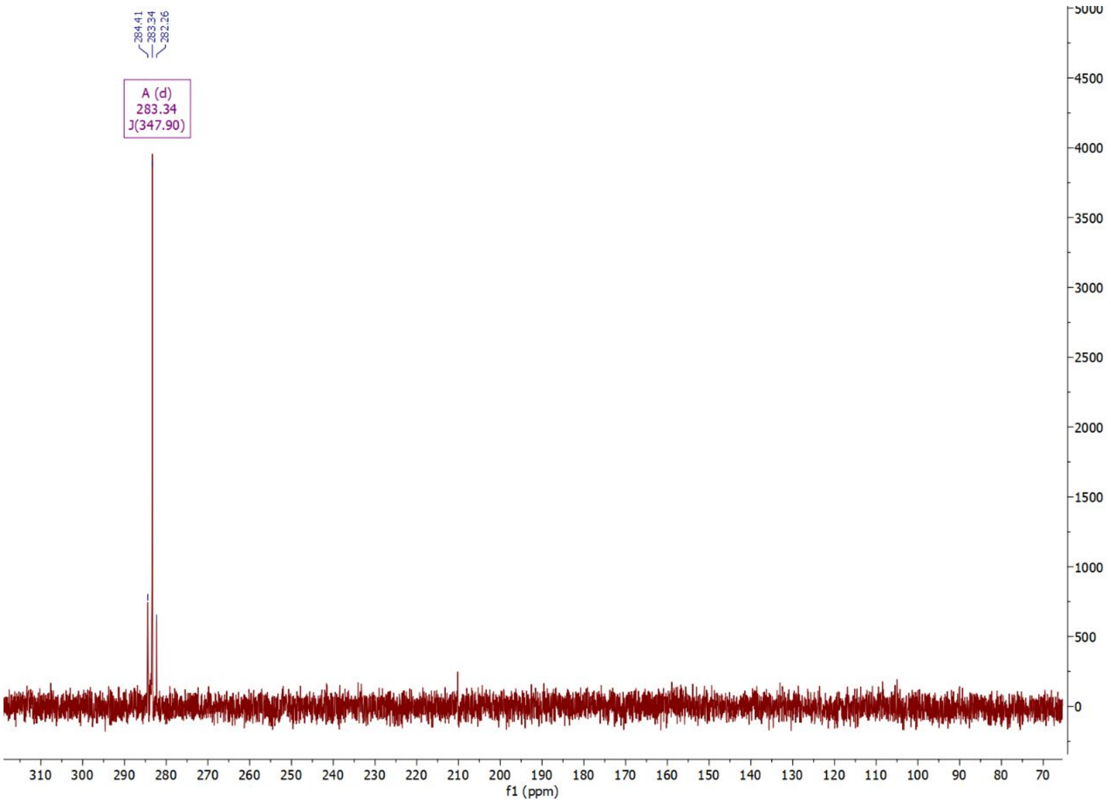

Figure S12. $^{31}P\{^{1}H\}$ NMR spectrum of compound **5** (C_6D_6).

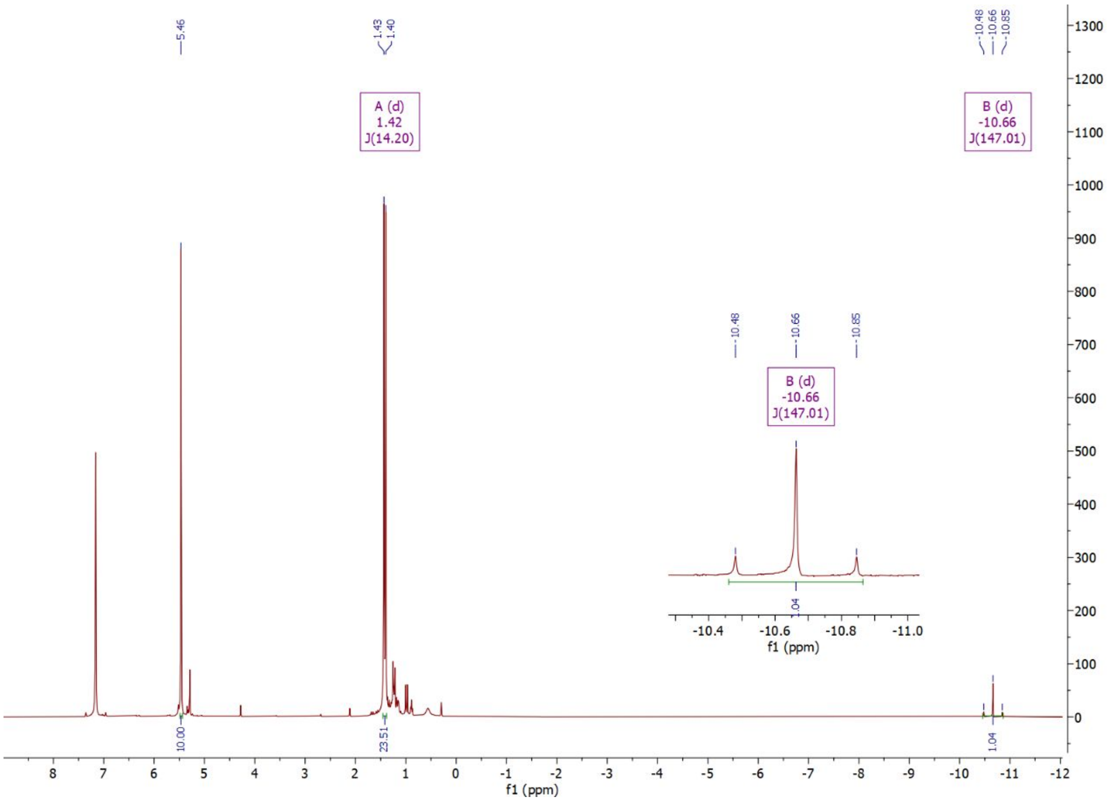

Figure S13. ^{1}H NMR spectrum of compound **5** (C_6D_6).

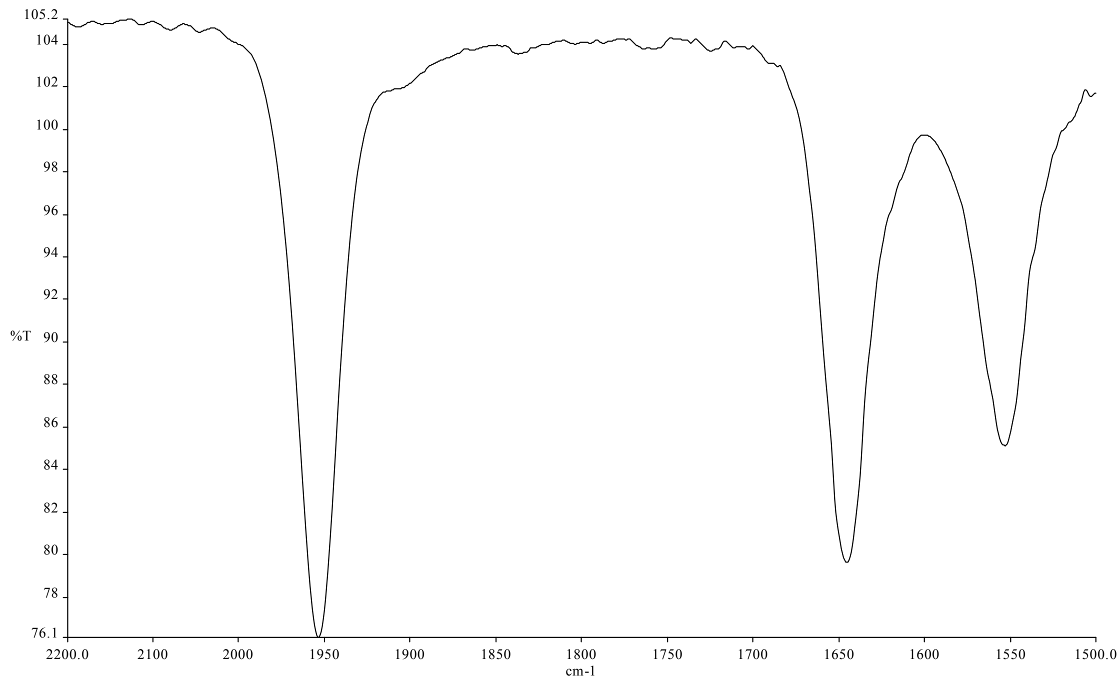

Figure S14. IR spectrum of compound **6** in dichloromethane solution.

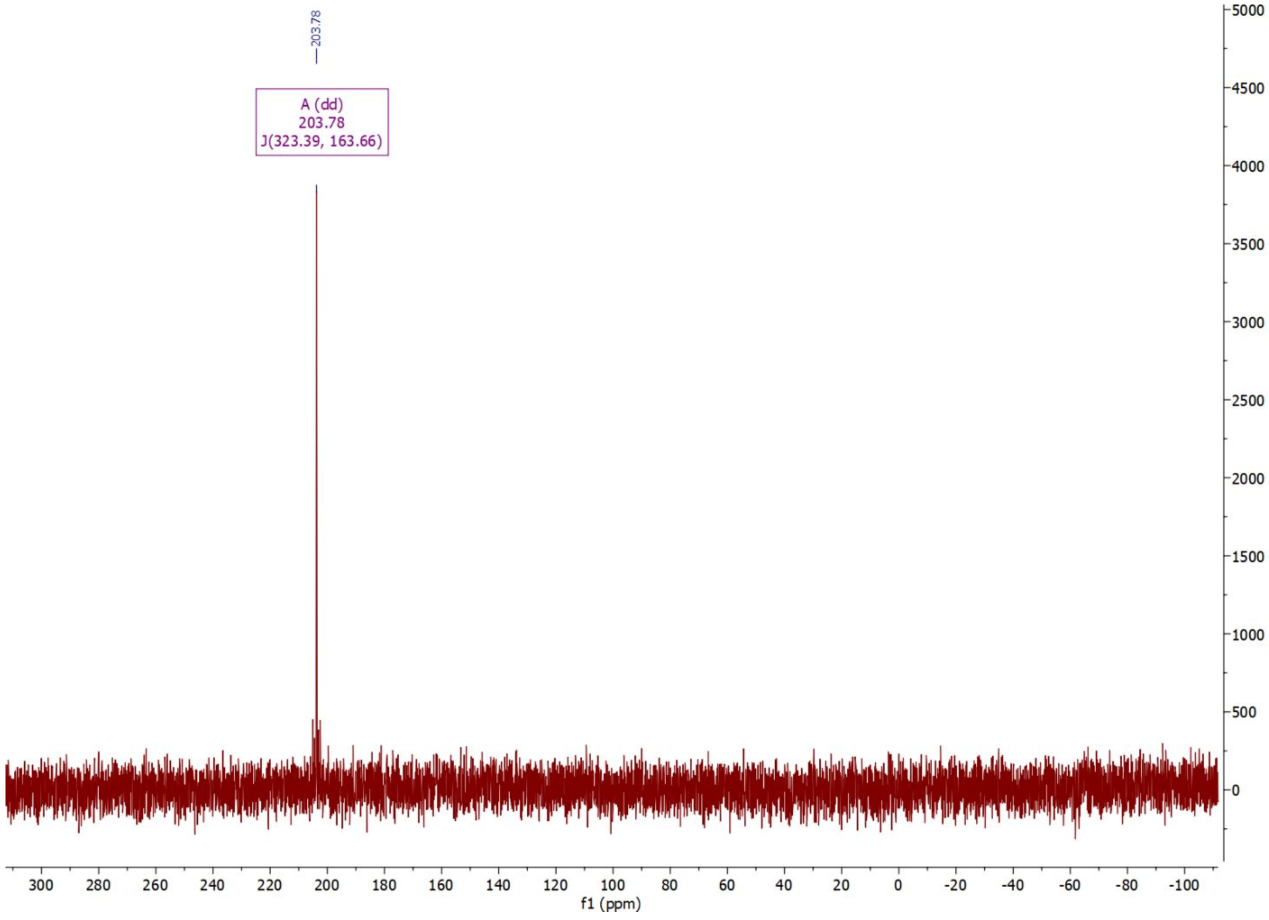

Figure S15. $^{31}P\{^{1}H\}$ NMR spectrum of compound **6** (CD_2Cl_2).

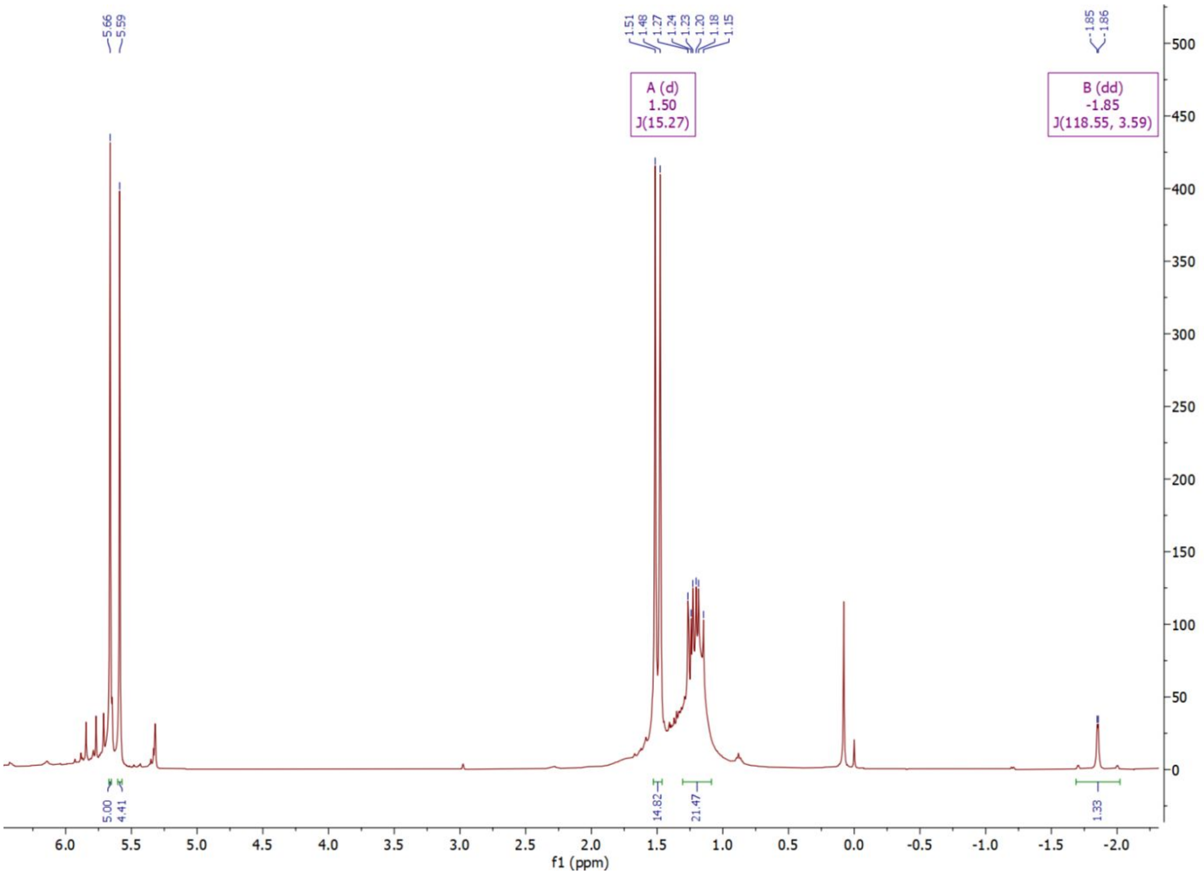

Figure S16. ^{1}H NMR spectrum of compound **6** (CD_2Cl_2, 295 K).

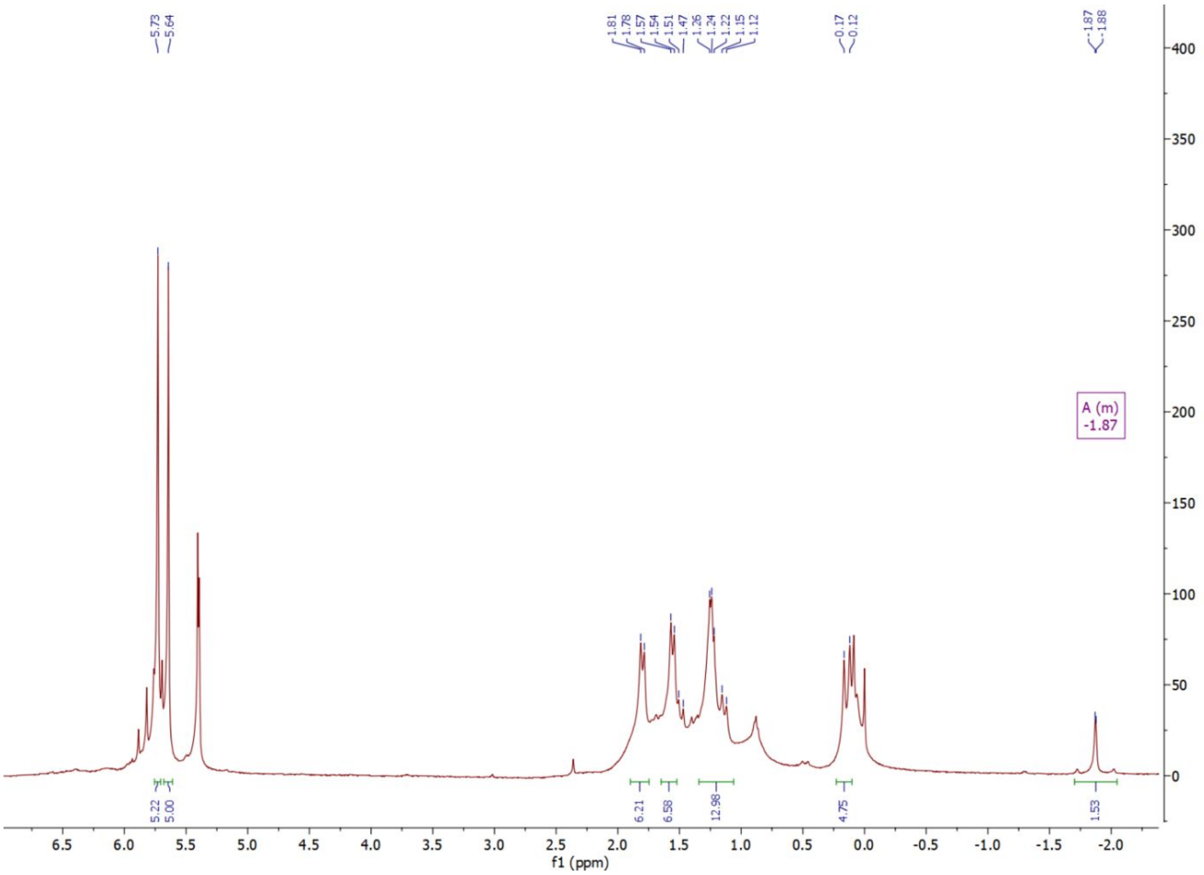

Figure S17. ^{1}H NMR spectrum of compound **6** (CD_2Cl_2. 223 K).

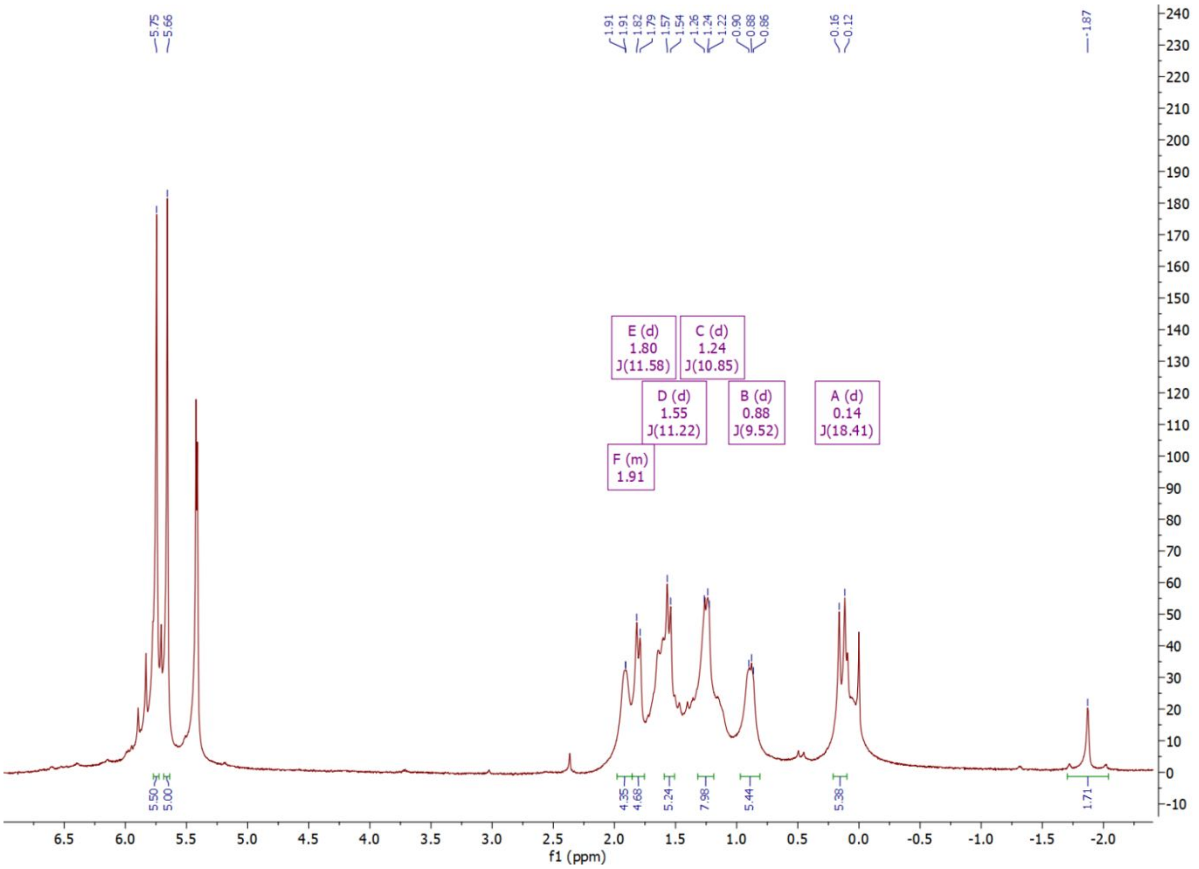

Figure S18. ^{1}H NMR spectrum of compound **6** (CD_2Cl_2. 193 K).

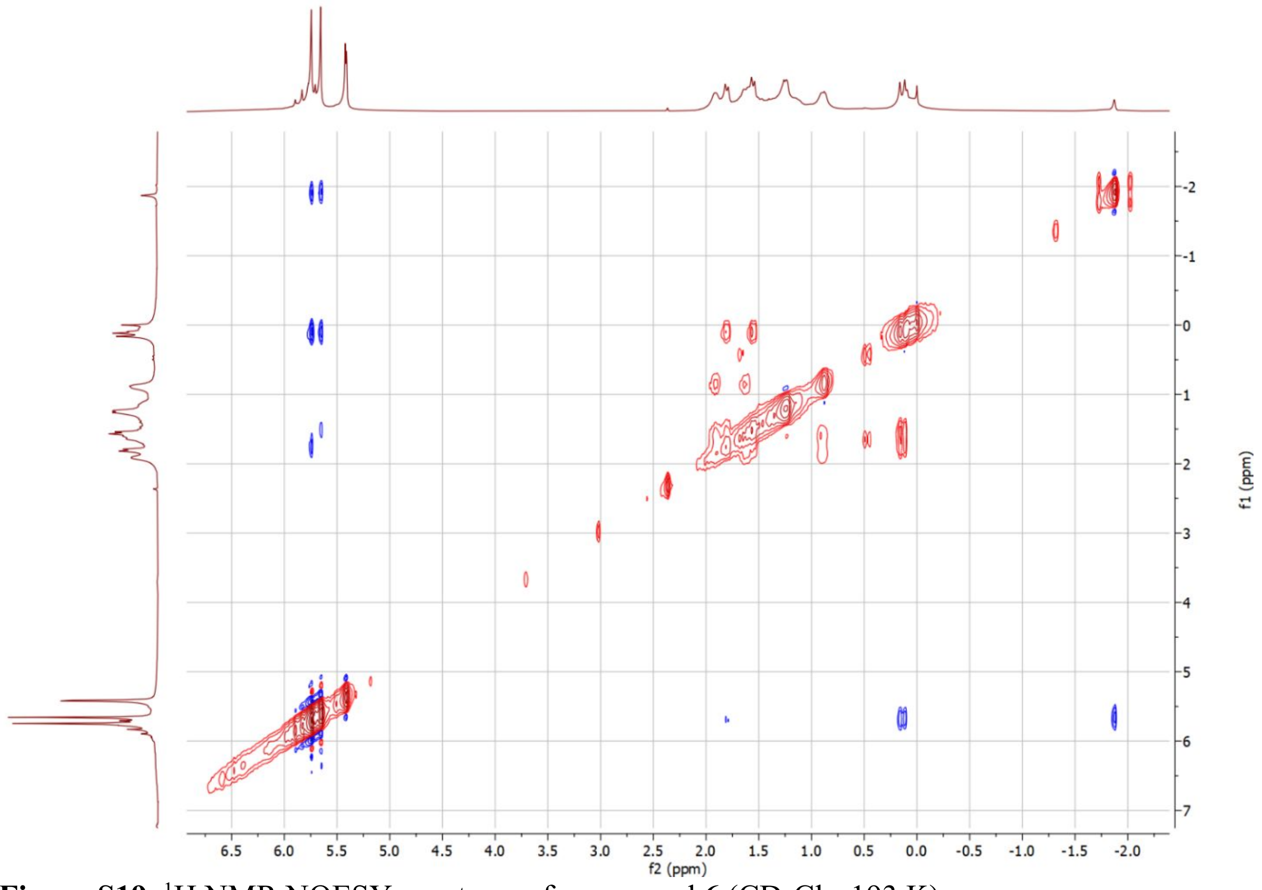

Figure S19. ^{1}H NMR NOESY spectrum of compound **6** (CD_2Cl_2. 193 K).

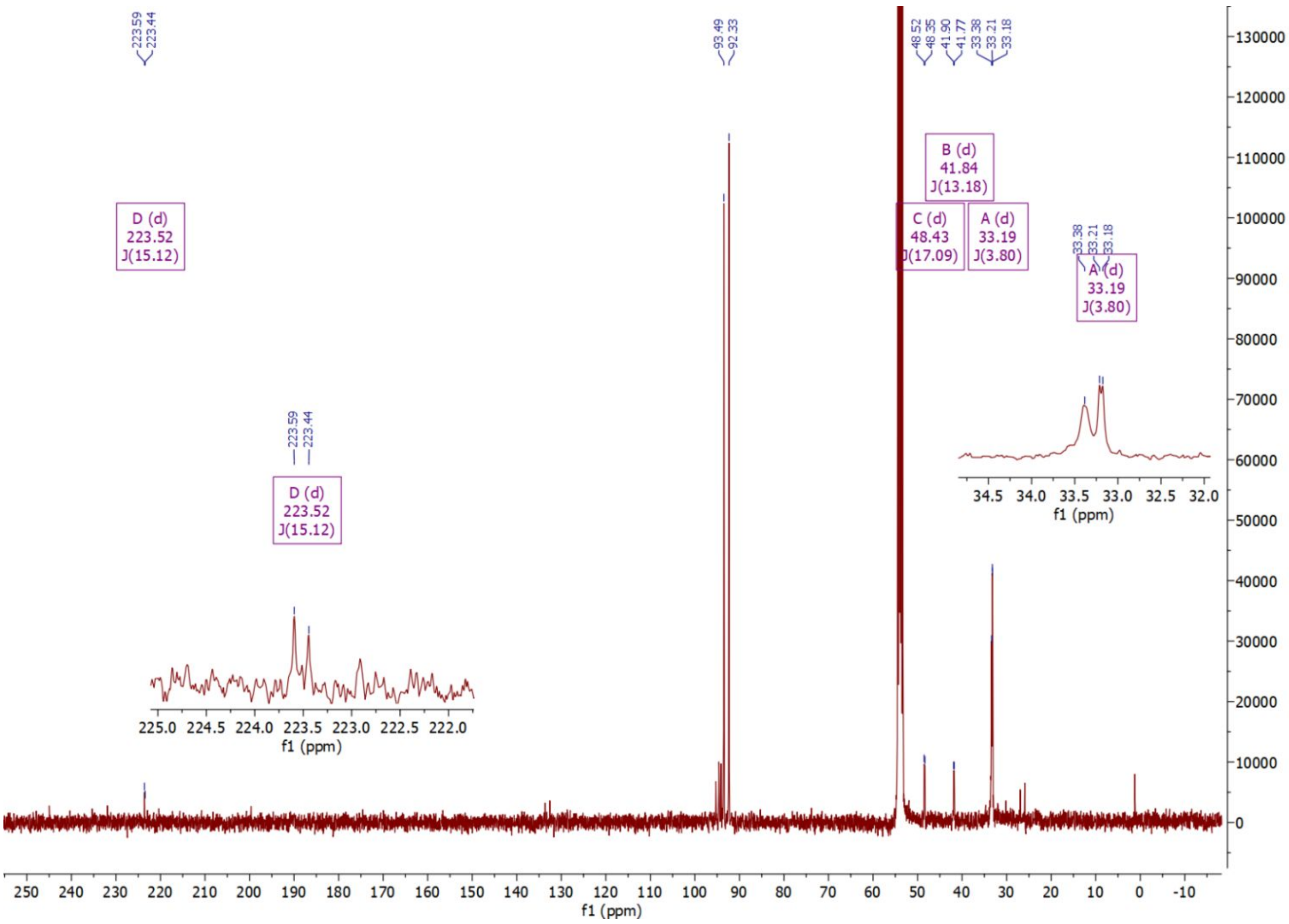

Figure S20. $^{13}C\{^{1}H\}$ NMR spectrum of compound **6** (CD_2Cl_2, 295 K).

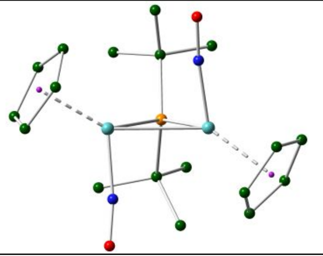	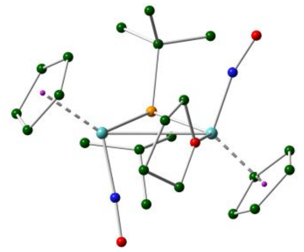
**T**+ S (0 / 2.570; 1.202)	**A**, (−2.0 / 2.993; 1.219)
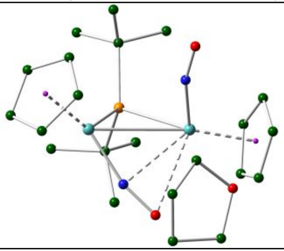	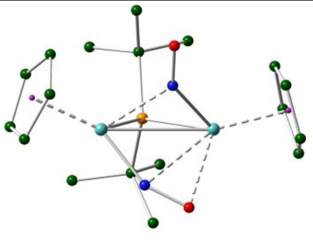
**TSA**, (+7.8 / 2.910; 1.238)	**I1**+ S (−1.7 / 2.896; 1.264)
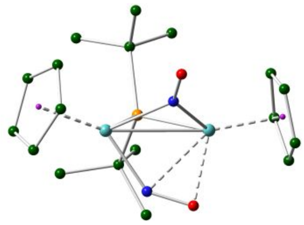	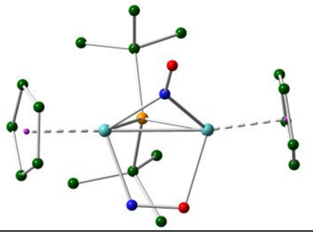
**TS1**+ S (+0.9 / 2.767; 1.263)	**I2**+ S (−6.2 / 2.599; 1.304)
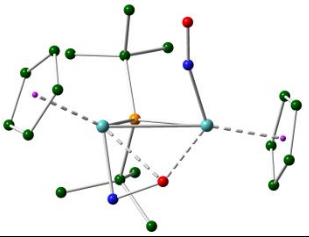	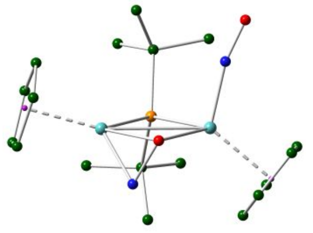
**TS2**+ S (+7.3 / 2.741; 1.406)	**I3**+ S (+0.6 / 2.830; 1.417)
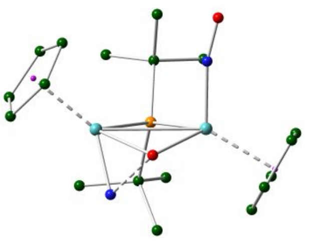	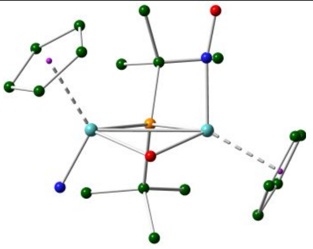
**TS3**+ S (+7.0 / 2.829; 1.698)	***trans*-2** + S (−45.3 / 2.884; 2.875)

Figure S21. M06L-DFT optimized structures for intermediates (**I****) and transition states (**TS****) involved in the rearrangement from radical[Mo$_2$Cp$_2$(μ-P^{t}Bu$_2$)(NO)$_2$] (**T**) to its most stable oxo-nitride isomer ***trans*-2**, with Gibbs free energies (in kcal/mol) relative to **T** + S, and Mo-Mo and N-O distances indicated between brackets (S = tetrahydrofuran).
